# Qualitative analysis of HIV and AIDS disease transmission: impact of awareness, testing and effective follow up

**DOI:** 10.12688/f1000research.123693.1

**Published:** 2022-10-07

**Authors:** Oluwakemi E. Abiodun, Olukayode Adebimpe, James Ndako, Olajumoke Oludoun, Benedicta Aladeitan, Michael Adeniyi

**Affiliations:** 1 Physical Sciences, Landmark University, Omu Aran, Kwara, 251101, Nigeria; 2 Mathematics and Statistic, First Technical University, Ibadan, Nigeria, 200103, Nigeria; 3 Mathematics and Statistic, Lagos State Polytechnic, Lagos, Lagos, Nigeria

**Keywords:** HIV/AIDS, infection-free equilibrium, defaulter lost to follow-up, endemic equilibrium, next generation matrix, basic reproduction number, stability.

## Abstract

**Background:** Since the early 1980s, human immunodeficiency virus (HIV) and its accompanying acquired immunodeficiency syndrome (AIDS) have spread worldwide, becoming one of the world's major global health issues. From the beginning of the epidemic until 2020, about 79.3 million people became infected, with 36.3 million deaths due to AIDS illnesses. This huge figure is a result of those unaware of their status due to stigmatization and invariably spreading the virus unknowingly.

**Methods:** Qualitative analysis through a mathematical model that will address HIV unaware individuals and the effect of an increasing defaulter on the dynamics of HIV/AIDS was investigated. The impact of treatment and the effect of inefficient follow-up on the transmission of HIV/AIDS were examined. The threshold for the effective reduction of the unaware status of HIV through testing, in response to awareness, and the significance of effective non-defaulting in treatment commonly called defaulters loss to follow-up as these individuals contribute immensely to the spread of the virus due to their increase in CD4+ count was determined in this study. Stability analysis of equilibrium points is performed using the basic reproduction number $R_0$, an epidemiological threshold that determines disease eradication or persistence in viral populations. We tested the most sensitive parameters in the basic reproduction numbers. The model of consideration in this study is based on the assumption that information (awareness) and non-stigmatization can stimulate change in the behaviours of infected individuals, and can lead to an increase in testing and adherence to treatment. This will in turn reduce the basic reproduction number, and consequently, the spread of the virus.

**Results:** The results portray that the early identification and treatment are inadequate for the illness to be eradicated.

**Conclusions:** Other control techniques, such as treatment adherence and effective condom usage, should be investigated in order to lessen the disease's burden.

## 1. Introduction

Human immunodeficiency virus (HIV) is a sexually transmitted infection (STI) and a blood-borne illness in humans with a wide range of clinical manifestations. ^1^^,^^2^ HIV and its accompanying acquired immune deficiency syndrome (AIDS) have spread rapidly around the world since its discovery in the early 1980s, and it remains the world’s most serious global health and development challenge. There is, however, a global devotion to avoiding new infections and making sure that all patients diagnosed have access to treatment. In addition, 79.3 million individuals have been infected with HIV since the pandemic began, with 36.3 million people dying due to AIDS diseases. About five million individuals contracted HIV for the first time in 2003, the largest number in any one year since the pandemic began. ^3^ Globally, the figure of persons living with HIV/AIDS has risen from 35 million in 2001 to 37.7 million in 2020, with around 3 million people dying from the illness in that year. ^4^^,^^5^ Around 84 percent [68 − 98 percent] of HIV-positive persons in the globe know their status in 2020, the remaining 16 percent (about 6 million people) [4.8 million-7.1 million] need to be tested for HIV. HIV testing is an important initial step in HIV prevention, treatment, care, and support. ^6^^,^^7^ Under Sustainable Development Goal 3, the international community pledged to work to end the AIDS pandemic by 2030. While progress has been made, it has been inconsistent, and the intermediate targets of “90-90-90” have been missed. ^7^^,^^8^ New diseases continue to wreak havoc on communities and undermine vital socioeconomic infrastructure all across the planet. According to the United Nations Joint Program on HIV and AIDS, the number of HIV-positive people in 2021 was 37.6 million, up from 33.2 million in 2010. ^9^ 1.5 million [1.1 million-2.1 million] people contracted HIV for the first time in 2020, 690,000 [480,000-1 million] people died of AIDS-related illnesses, and antiretroviral medication was available to 27.4 million [26.5 million-27.7 million] patients in December 2020, up from 7.8 million [6.9 million-7.9 million] in 2010. ^9^^–^^11^ HIV can be spread horizontally or vertically from one infected individual to another. Horizontal HIV transmission occurs when an individual comes into direct contact with an HIV-positive person, including sexual contact, or when they use a needle and syringe that has recently been utilized by a HIV-positive individual. Contrastingly, vertical transmission occurs when the virus is passed directly from an infected mother to her pregnant or newborn child. ^12^ HIV/AIDS transmission dynamics has piqued the interest of applied mathematicians, epidemiologists ^13^^–^^16^ and biologists ^17^^–^^22^ due to the disease’s worldwide menace. Various improvements have been made to May and Anderson’s early models, ^23^^–^^25^ and particular issues have been discussed by researchers. ^12^^,^^26^^–^^48^ In Lu *et al*. 2020 ^27^ fostered a compartmental model for the yearly revealed HIV/AIDS MSM in the Zhejiang Region of China between 2007 to 2019 and anticipated that 90 percent of people tested for HIV/AIDS will have received treatment by 2020, while the screened extent will remain as low as 40 percent, and that antiretroviral treatment (ART) can actually control the transmission of HIV, even within the sight of medication opposition. In Rana and Sharma, 2020 ^30^ presented a simple Likely to be exposed-Infected (i.e.SI) form of HIV/AIDS mathematical model, in view of the supposition that changing from an AIDS-infected to an HIV-infected individual is conceivable, in order to understand disease dynamics and develop strategies to reduce or control disease transmission among individual. Mushanyu ^32^ built a mathematical model for HIV acquisition using nonlinear ordinary differential equations to analyse the influence of delayed HIV diagnosis on the transmission of HIV in the year 2020. To prevent HIV from spreading further, the researchers advocated for early HIV treatment and the expansion of HIV self-testing initiatives, which would allow more people who have not been tested for HIV to learn their status. Teng ^12^ proposed and investigated a time-delay compartmental framework for HIV transmission in a sexually active cohort with press coverage, a disease that can result to a developed phase of infection known as acquired immunodeficiency syndrome (AIDS), as well as vertical transmission in the enrollment of people infected in 2019. ^33^ Saad *et al*. (2019) developed and considered an *HIV* ^+^ mathematical model with the next generation matrix, the infection-free and endemic equilibrium points were identified, and the basic reproduction ratio *R*_0_ was determined. The Lyapunov function was utilized to analyze the equilibria’s global stability, and it was observed that the equilibria’s stability is reliant on the magnitude of the fundamental reproduction ratio. ^37^ developed an HIV/AIDS epidemic model with a generic nonlinear rate of occurrence and therapy, was able to obtain the basic reproductive number *R*_0_ using the next generating matrix technique.

Researchers have employed numerous tools to manage and eradicate HIV/AIDS diseases. ^3^^,^^11^^,^^12^ These studies revealed that awareness creation/information can help to control the disease burden but cannot eliminate the disease. Furthermore, there are other techniques and tools available that can be applied to study the dynamics of disease transmission and to provide suitable control interventions. The use of mathematical modeling is foremost among these techniques. ^16^^–^^19^ Although many articles ^20^ have studied the impact of different controls; however, none of them have incorporated human behavior in response to information. Hence, this study identifies the threshold for effective reduction of HIV/AIDS, as a result of HIV unaware individuals and consequent effective follow up in the use treatment.

The following is the structure of the paper: Section 2 describes the model, while Section 3 examines the model’s basic features, the basic reproduction number, and equilibrium points. Section 4 employs parameter sensitivity index on the reproduction number to conduct a stability study of the equilibria (local and global), and the findings are generated from numerical simulations of data from previously published studies in Section 5. Finally, the research is examined and completed in Section 6.

## 2. Model formulation and description

**Figure 1.**
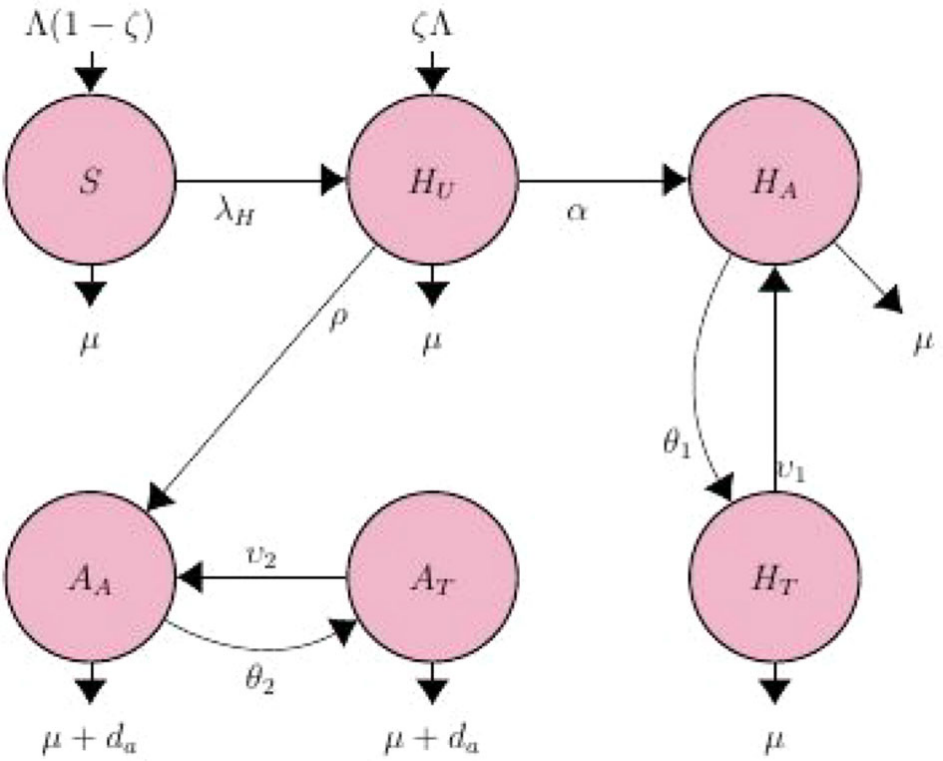
HIV/AIDS compartmental flow diagram.

A mathematical model on the mechanisms of horizontal and vertical transmission of HIV/AIDS was developed, by incorporating the effect of testing, defaulter lost to follow-up on treatment, and effective use of condom on the existing model. The model is available from GitHub and is archived with Zenodo. ^66^ The model is depicted schematically in Figure 1. The model contains six (6) state variables, namely: Susceptible, ( *S*), representing people who are likely to become infected with HIV; Unaware HIV infectives, ( *H _U_*), Aware HIV infectives ( *H _A_*), Treated HIV infectives, ( *H _T_*); AIDS individuals ( *A _A_*) and AIDS on treatment individuals ( *A _T_*). The rate of effective contact with HIV-positive people either by immigration or emigration is given by Λ. A percentage of newborns get infected with HIV during birth at a rate of (1 − *ζ*) and are therefore directly enrolled into the unaware infected population *H _U_*, at a rate *ζ*Λ, with 0 ≤ *ζ* ≤ 1. $\lambda_{H}=c\left(1-\psi \xi \right)\frac{\beta_{1}H_{U}+\beta_{2}H_{A}+\beta_{3}A_{A}}{N}$ is the HIV transmission contact rate. Parameter *c* represents the average number of sexual partners acquired by people who is vulnerable to HIV annually. In order to simulate the influence of condom usage as a significant preventive intervention, the amount of condom protection (usage and effectiveness) is given as *ψξ*[0, 1] based on assumption. If *ξ* = 0, condom use provides no protection, but *ξ* = 1 denotes complete protection, where *ψ* is the condom use. The parameters *β*_1_, *β*_2_ and *β*_3_ account for the HIV transfer rates between persons at risk and (HIV unaware, HIV aware and full blown AIDS) infectives individuals, respectively. Both the HIV-infected and the AIDS-infected groups are thought to be active in the spread of HIV/AIDS amongst susceptible. Because infected patients with AIDS symptoms have a greater viral load than HIV positive people (pre-AIDS) in the *H _U_ and H _A_* classes, and because viral load and infectiousness have a positive connection, we must have *β*_1_ < *β*_2_ < *β*_3_. There is an evidence to suggest that individuals who know their HIV status *H _A_* change their sexual behavior (i.e. adopt safer-sex practices), resulting in reduced transmission. ^25^ Most HIV pandemic models disregard the role of AIDS patients in HIV transmission by applying simplistic assumptions such as AIDS death being immediate or AIDS patients being incapable of mingling and gaining new sex partners. However, epidemiological data shows that AIDS patients participate in hazardous sexual activities, such as seldom wearing condoms or having several sex partners. ^61^ As shown in the findings of ^21^ a research of HIV-1-infected transfusion men and their women sex partners, severe AIDS patients are more likely to infect their partners than non-advanced immuno-compromised receivers. ^62^ also reported similar findings. HIV-positive individuals with and without AIDS signs are likely to have access to antiretroviral therapy (ART). Unaware HIV-infected persons, *H _U_*, progress to the category of aware HIV infection *H _A_*, after testing at a rate of *α*, while unaware infected individual who did not go for testing progress to stage IV of AIDS, *A _A_*; at a rate of *ρ.* HIV-infected aware people with no symptoms of AIDS; *H _A_*, proceed to the group of HIV infection under ART therapy, *H _T_*, whereas HIV-infected people with AIDS symptoms, *A _A_*, are treated for AIDS at a rate of *θ*_2_ on reaching the class of *A _T_.* We presume that HIV-infected people on treatment do not spread the virus. ^49^^,^^50^ HIV-infected people who are receiving therapy but do not have AIDS symptoms, *H _T_*, who default during treatment and become resistant to drug, will return to the HIV-infected aware individuals, *H _A_*, and that HIV-infected persons with AIDS symptoms, *A _A_*, who default during treatment in class *A _T_*, become re-infected with HIV with symptoms of AIDS individuals, *A _A_*, at a rate *υ*_1_ and *υ*_2_ respectively. ^51^ It is assumed that only HIV-infected people with AIDS symptoms, *A _A_* and *A _T_*, die of AIDS-related causes at a rate of *d _a_.* The following mathematical model is based on these assumptions and that the system has a natural death in each class at a rate *μ.*

In order to contribute to the arduous aim of ending it by 2030 there is need to foresee the epidemic’s behaviour. One of the most significant tools we’ll utilize to attain our aim is mathematical modeling of HIV infection. Based on, ^52^ the following model was developed by the inclusion of AIDS on treatment compartment (by considering treatment of both individual not showing and showing symptoms of AIDS), individual who fall-out of treatment, considering AIDS individual are able to transmit infection, condom use to control transmission rate and average number of sexual partners acquired on force of infection. A system of ordinary differential equations (ODEs) can be used to express the mathematical equations that correspond to the schematic diagram:

$$\begin{array}{l} \frac{\mathrm{dS}}{\mathrm{dt}}=\Lambda \left(1-\zeta H_{U}\right)-\left(\lambda_{H}+\mu \right)S\;\left(a\right)\\ \frac{{\mathrm{dH}}_{U}}{\mathrm{dt}}=\Lambda \zeta H_{U}+\lambda_{H}S-\left(\alpha +\rho +\mu \right)H_{U}\;\left(b\right)\\ \frac{{\mathrm{dH}}_{A}}{\mathrm{dt}}=\alpha H_{U}+v_{1}H_{T}-\left(\theta_{1}+\mu \right)H_{A}\;\left(c\right)\\ \frac{{\mathrm{dH}}_{T}}{\mathrm{dt}}=\theta_{1}H_{A}-\left(v_{1}+\mu \right)H_{T}\;\left(d\right)\\ \frac{{\mathrm{dA}}_{A}}{\mathrm{dt}}=\rho H_{U}+v_{2}A_{T}-\left(\theta_{2}+d_{a}+\mu \right)A_{A}\;\left(e\right)\\ \frac{{\mathrm{dA}}_{T}}{\mathrm{dt}}=\theta_{2}A_{A}-\left(v_{2}+d_{a}+\mu \right)A_{T}\;\left(f\right) \end{array}$$ (1)

with the positive initial conditions given as:

$$S\left(0\right)=S_{0},H_{U}\left(0\right)=H_{U0},H_{A}\left(0\right)=H_{A0},H_{T}\left(0\right)=H_{T0},A_{A}\left(0\right)=A_{A0},A_{T}\left(0\right)=A_{T0}$$ (2)

## 3. Model investigation

### 3.1 Region of invariant

All of the parameters in the model are considered to be non-negative. System (1), on the other hand, keeps track of the human populace, hence, the state variables are always positive for all time *t* ≥ 0. Thus, the total human populace is given as

$$N\left(t\right)=S\left(t\right)+H_{U}\left(t\right)+H_{A}\left(t\right)+H_{T}\left(t\right)+A_{A}\left(t\right)+A_{T}\left(t\right)$$ (3)

Here equation (1) is changing at a rate

$$\frac{\mathrm{dN}}{\mathrm{dt}}=\frac{\mathrm{dS}}{\mathrm{dt}}+\frac{{\mathrm{dH}}_{U}}{\mathrm{dt}}+\frac{{\mathrm{dH}}_{A}}{\mathrm{dt}}+\frac{{\mathrm{dH}}_{T}}{\mathrm{dt}}+\frac{{\mathrm{dA}}_{A}}{\mathrm{dt}}+\frac{{\mathrm{dA}}_{T}}{\mathrm{dt}}=\Lambda -\mathrm{\mu N}-d_{a}A_{A}-d_{a}A_{T}+\varphi H_{U}$$ (4)

In the non-existence of infection i.e for *H _U_* = *H _A_* = *H _T_* = *A _A_* = *A _T_* = 0 we have,

$$\frac{\mathrm{dN}}{\mathrm{dt}}\leq \Lambda -\mu$$ (5)

We must have (6) by separating the variables of differential inequality.

$$\frac{\mathrm{dN}}{\Lambda -\mathrm{\mu N}}\leq \mathrm{dt}$$ (6)

Integrating the above equation we have

$$\Lambda -\mathrm{\mu N}\geq {\mathrm{Ce}}^{-\mathrm{\mu t}}$$

where *C* is a constant to which to be determined. Let at *t* = 0, *N* = *N*_0_. So we have,

$$C=\Lambda -\mu N_{0}$$ (7)

From (7) we have

$$\begin{array}{l} \Lambda -\mathrm{\mu N}\geq \left(\Lambda -\mu N_{0}\right)e^{-\mathrm{\mu t}}\\ \Rightarrow N\left(t\right)\leq \frac{\Lambda }{\mu }-\left[\frac{\Lambda -\mu N_{0}}{\mu }\right]e^{-\mathrm{\mu t}} \end{array}$$

As $t\to \infty ,0\leq N\left(t\right)\leq \frac{\Lambda }{\mu }$

As a result, the system (1) feasible solutions set enters the region.

$$\Omega =\left\{\left(S,H_{U},H_{A},H_{T},A_{A},A_{T}\right)\in {ℛe}_{+}^{6}:0\leq N\leq \frac{\Lambda }{\mu }\right\}$$

when $N\leq \frac{\Lambda }{\mu }$ every solution with an initial condition in ${ℛe}_{+}^{6}$ stays in that region for *t* > 0. As a result, the model is well posed and epidemiologically relevant in the domain Ω.

### 3.2 Non-negativity of solutions

This section discusses the positivity of the solutions, which describes the system’s non-negativity of solutions (1).

**Lemma 1:** *S*( *t*) ≥ 0, *H _U_* ( *t*) ≥ 0, *H _A_* ( *t*) ≥ 0, *H _T_* ( *t*) ≥ 0, *A _A_* ( *t*) ≥ 0, *A _T_* ( *t*) ≥ 0 and *N*( *t*) ≥ 0 satisfied by the solutions of system (1) with initial conditions (2) for all *t* ≥ 0. The region $\Omega \subset ℛ\rceil_{+0}^{6}$ is positively invariant and attracts in terms of system (1).

**Proof:** Take a look at the first equation in (1)

$$\frac{\mathrm{dS}}{\mathrm{dt}}=\Lambda \left(1-\zeta H_{U}\right)-\left(\lambda_{H}+\mu \right)S$$

we have;

$$\frac{\mathrm{dS}}{\mathrm{dt}}\geq -\left(\Lambda \zeta H_{U}+\lambda_{H}+\mu \right)S\int \frac{1}{S}\mathrm{dS}\int -\left(\Lambda \zeta H_{U}+\lambda_{H}+\mu \right)\mathrm{dt}$$

$$S\geq S_{0}e^{-\left(\Lambda \zeta H_{U}+\lambda_{H}+\mu \right)}\geq 0$$

provided $\left(\Lambda \zeta H_{U}+\lambda_{H}+\mu \right)<\infty$

As a result, *S* ≥ 0

Likewise, for system (1)’s second equation, we have

$$\frac{{\mathrm{dH}}_{U}}{\mathrm{dt}}=\Lambda \zeta H_{U}+\lambda_{H}S-\left(\alpha +\rho +\mu \right)H_{U}$$

$$\frac{{\mathrm{dH}}_{U}}{\mathrm{dt}}\geq -\left(\alpha +\rho +\mu \right)H_{U}\int \frac{1}{H_{U}}{\mathrm{dH}}_{U}\int -\left(\alpha +\rho +\mu \right)\mathrm{dt}$$

$$H_{U}\geq H_{U0}e^{-\left(\alpha +\rho +\mu \right)}\geq 0$$

provided $\left(\alpha +\rho +\mu \right)<\infty$

Hence, *H _U_* ≥ 0

similarly it can be shown that *H _A_* ≥ 0, *H _T_* ≥ 0, *A _A_* ≥ 0, *A _T_* ≥ 0 for all *t* > 0

Thus the solutions *S*, *H _U_*, *H _A_*, *H _T_*, *A _A_*, *A _T_* remain positive forever.

### 3.3 Equilibrium point and basic reproduction number; *R*_0_

The model (1) has exactly one disease-free equilibrium (DFE) point and the equilibrium point *E*_0_ is given by $\left(S_{0},H_{U0},H_{A0},H_{T0},A_{A0},A_{T0}\right)=\left(\frac{\Lambda }{\mu },0,0,0,0,0\right).$ In the absence of infection, the total population changes in proportion to the ratio of recruitment rate to the death rate.

The total population dynamics can be altered when an individual with an HIV/AIDS is introduced into a population. For the endemic equilibrium, there is an existence of infection hence *H _U_* ≠ *H _A_* ≠ *H _T_* ≠ *A _A_* ≠ *A _T_* ≠0. It is denoted by E_*_. Setting equation (1a-1f) equal to zero which exist when *R*_0_ > 1 we have

$$S_{*}=\frac{\left(M_{1}-\zeta \Lambda \right)\left(M_{4}M_{5}-\upsilon_{2}\theta_{2}\right)}{\lambda \rho M_{5}}{A_{A}}^{*}$$ (8)

$$H_{U*}=\frac{\left(M_{4}M_{5}-\upsilon_{2}\theta_{2}\right)}{\rho M_{5}}{A_{A}}^{*}$$ (9)

$$H_{A*}=\frac{\alpha M_{3}\left(-\upsilon_{2}\theta_{2}+M_{4}M_{5}\right)}{\left(M_{2}M_{3}-\upsilon_{1}\theta_{1}\right)}{A_{A}}^{*}$$ (10)

$$H_{T*}=\frac{\theta_{1}\alpha M_{3}\left(\upsilon_{2}\theta_{2}-M_{4}M_{5}\right)}{M_{3}\left(M_{2}M_{3}-\upsilon_{1}\theta_{1}\right)}{A_{A}}^{*}$$ (11)

$$A_{A*}=\frac{\Lambda \rho M_{5}\lambda }{M_{1}\left(M_{4}M_{5}-\upsilon_{2}\theta_{2}\right)\lambda +\mu \left(M_{1}-\zeta \Lambda \right)\left(M_{4}M_{5}-\upsilon_{4}\theta_{2}\right)}$$ (12)

$$A_{T*}=\frac{\theta_{2}}{M_{5}}A_{A*}$$ (13)

*M*_1_ = *α* + *ρ* + *μ*, *M*_2_ = *θ*_1_ + *μ*, *M*_3_ = *υ*_1_ + *μ*, *M*_4_ = *θ*_2_ + *d _a_* + *μ*, *M*_5_ = *υ*_2_ + *d _a_* + *μ*.

**Theorem 1:** There exists a positive endemic equilibrium if *R*_0_ > 1

Reference 53 presented a better method for determining *R*_0_ which was an improved technique of solving the reproduction number firstly developed by Ref. 54 that is widely accepted because it represents the biological meaning of *R*_0_. By considering only the infective classes, we were able to obtain the system’s (1) basic reproduction number, *R*0, which is the spectral radius ( *ρ*) of the next generation matrix, *NGM*, i.e. $R_{0}=\rho \left({\mathrm{FV}}^{-1}\right)$ . The rate of emergence of new infections in compartments *i*, while *V* denotes the rate of transfer of individual into and out of the compartment *i* by all other means. Where F and V are the *m* × *m* matrices defined as:

$F=\frac{\partial F_{i\left(\mathrm{xo}\right)}}{\partial_{\mathrm{xj}}}\;\text{and}\;V=\frac{\partial V_{i\left(\mathrm{xo}\right)}}{\partial_{\mathrm{xj}}}\;\text{with}\;i\leq i,j\leq m$

*F* is non-negative and *V* is non-singular matrix.

Then,

$$F=\left(\begin{array}{ccccc} c\left(1-\psi \xi \right)\beta_{1} & c\left(1-\psi \xi \right)\beta_{2} & 0 & c\left(1-\psi \xi \right)\beta_{3} & 0\\ 0 & 0 & 0 & 0 & 0\\ 0 & 0 & 0 & 0 & 0\\ 0 & 0 & 0 & 0 & 0\\ 0 & 0 & 0 & 0 & 0 \end{array}\right)\text{and}\text{V}=\left(\begin{array}{ccccc} M_{1}-\Lambda \zeta & 0 & 0 & 0 & 0\\ -\alpha & M_{2} & -\upsilon_{1} & 0 & 0\\ 0 & -\theta_{1} & M_{3} & 0 & 0\\ -\rho & 0 & 0 & M_{4} & -\upsilon_{2}\\ 0 & 0 & 0 & -\theta_{2} & M_{5} \end{array}\right)FV^{-1}=\left[\begin{array}{ccccc} \frac{c\left(1-\psi \xi \right)\beta_{1}}{\Lambda \zeta -M_{1}}+\frac{c\left(1-\psi \xi \right)\beta_{2}\alpha M_{3}}{\Lambda \zeta M_{2}M_{3}-\Lambda {\zeta \theta }_{1}\upsilon_{1}-M_{1}M_{2}M_{3}+M_{1}\theta_{1}\upsilon_{1}}+\frac{c\left(1-\psi \xi \right)\beta_{3}\rho M_{4}}{\Lambda \zeta M_{4}^{2}-\Lambda {\zeta \theta }_{2}\upsilon_{2}-M_{1}k_{4}^{2}+M_{1}\theta_{2}\upsilon_{2}} & \frac{c\left(1-\psi \xi \right)\beta_{2}M_{3}}{M_{2}M_{3}-\theta_{1}\upsilon_{1}} & \frac{c\left(1-\psi \xi \right)\beta_{2}\upsilon_{1}}{M_{2}M_{3}-\theta_{1}\upsilon_{1}} & \frac{c\left(1-\psi \xi \right)\beta_{3}M_{4}}{M_{4}^{2}-\theta_{2}\upsilon_{2}} & \frac{c\left(1-\psi \xi \right)\beta_{3}\upsilon_{2}}{M_{4}^{2}-\theta_{2}\upsilon_{2}}\\ 0 & 0 & 0 & 0 & 0\\ 0 & 0 & 0 & 0 & 0\\ 0 & 0 & 0 & 0 & 0\\ 0 & 0 & 0 & 0 & 0 \end{array}\right]$$ (14)

where *M*_1_ = *α* + *μ* + *ρ*, *M*_2_ = *θ*_1_ + *μ*, *M*_3_ = *v*_1_ + *μ*, *M*_4_ = *θ*_2_ + *d _a_* + *μ*, *M*_5_ = *v*_2_ + *d _a_* + *μ*

The model reproduction number, denoted by *R*_0_ is thus given by $R_{0}=\rho \left({\mathrm{FV}}^{-1}\right)=R=R_{1}+R_{2}+R_{3}$ , the spectral radius of the NGM *FV* ^−1^.

Here,

$$\begin{array}{l} R_{1}=\frac{c\left(1-\psi \xi \right)\beta_{1}}{\zeta \Lambda -M_{1}}\\ R_{2}=\frac{c\left(1-\psi \xi \right)\beta_{2}\alpha M_{3}}{\left(M_{2}M_{3}-\theta_{1}\upsilon_{1}\right)\left(\zeta \Lambda -M_{1}\right)}\\ R_{3}=\frac{c\left(1-\psi \xi \right)\beta_{3}\rho M_{4}}{\left(M_{4}^{2}-\theta_{2}\upsilon_{2}\right)\left(\zeta \Lambda -M_{1}\right)} \end{array}$$

## 4. Equilibria stability analysis

### 4.1 Disease-free equilibrium stability on a local and global scale, *E*_0_

**Theorem 2:** For all *R*_0_, the disease-free equilibrium *E*_0_ exists, and it is locally asymptotically stable for *R*_0_ < 1 and unstable otherwise.

**Proof:** The resulting matrix from linearized model $\frac{\mathrm{dx}}{\mathrm{dt}}=\mathrm{AX},$ where $X={\left(x_{1},x_{2},x_{3},x_{4},x_{5},x_{6}\right)}^{T},\left(x_{1},x_{2},x_{3},x_{4},x_{5},x_{6}\right)\in R_{+}^{6}$ , and

$$A=\left(\begin{array}{cccccc} g1-\mu & g2-\Lambda \zeta & g5 & \frac{c\left(1-\psi \xi \right)\left(\beta_{2}H_{A}+\beta_{3}A_{A}+H_{U}\beta_{1}\right)S}{{\left(S+H_{U}+H_{A}+H_{T}+A_{A}+A_{T}\right)}^{2}} & g7 & \frac{c\left(1-\psi \xi \right)\left(\beta_{2}H_{A}+\beta_{3}A_{A}+H_{U}\beta_{1}\right)S}{{\left(S+H_{U}+H_{A}+H_{T}+A_{A}+A_{T}\right)}^{2}}\\ g3 & \zeta \Lambda -\alpha +g4-\mu -\rho & g6 & -\frac{c\left(1-\psi \xi \right)\left(\beta_{2}H_{A}+\beta_{3}A_{A}+H_{U}\beta_{1}\right)S}{{\left(S+H_{U}+H_{A}+H_{T}+A_{A}+A_{T}\right)}^{2}} & g8 & -\frac{c\left(1-\psi \xi \right)\left(\beta_{2}H_{A}+\beta_{3}A_{A}+H_{U}\beta_{1}\right)S}{{\left(S+H_{U}+H_{A}+H_{T}+A_{A}+A_{T}\right)}^{2}}\\ 0 & \alpha & -\theta_{1}-\mu & \upsilon_{1} & 0 & 0\\ 0 & 0 & \theta_{1} & -\upsilon_{1}-\mu & 0 & 0\\ 0 & \rho & 0 & 0 & -\theta_{2}-d_{a}-\mu & \upsilon_{2}\\ 0 & 0 & 0 & 0 & \theta_{2} & -\upsilon_{2}-d_{a}-\mu \end{array}\right)$$ (15)

$$g_{1}=\frac{c\left(1-\psi \xi \right)\left(\beta_{2}H_{A}+\beta_{3}A_{A}+H_{U}\beta_{1}\right)S}{{\left(S+H_{U}+H_{A}+H_{T}+A_{A}+A_{T}\right)}^{2}}-\frac{c\left(1-\psi \xi \right)\left(\beta_{2}H_{A}+\beta_{3}A_{A}+H_{U}\beta_{1}\right)}{S+H_{U}+H_{A}+H_{T}+A_{A}+A_{T}},$$

$$g_{2}=\frac{c\left(1-\psi \xi \right)\left(\beta_{2}H_{A}+\beta_{3}A_{A}+H_{U}\beta_{1}\right)S}{{\left(S+H_{U}+H_{A}+H_{T}+A_{A}+A_{T}\right)}^{2}}-\frac{c\left(1-\psi \xi \right)\beta_{1}S}{S+H_{U}+H_{A}+H_{T}+A_{A}+A_{T}},$$

$$g_{3}=\frac{c\left(1-\psi \xi \right)\left(\beta_{2}H_{A}+\beta_{3}A_{A}+H_{U}\beta_{1}\right)}{S+H_{U}+H_{A}+H_{T}+A_{A}+A_{T}}-\frac{c\left(1-\psi \xi \right)\left(\beta_{2}H_{A}+\beta_{3}A_{A}+H_{U}\beta_{1}\right)S}{{\left(S+H_{U}+H_{A}+H_{T}+A_{A}+A_{T}\right)}^{2}},$$

$$g_{4}=\frac{c\left(1-\psi \xi \right)\beta_{1}S}{S+H_{U}+H_{A}+H_{T}+A_{A}+A_{T}}-\frac{c\left(1-\psi \xi \right)\left(\beta_{2}H_{A}+\beta_{3}A_{A}+H_{U}\beta_{1}\right)S}{{\left(S+H_{U}+H_{A}+H_{T}+A_{A}+A_{T}\right)}^{2}},$$

$$g_{5}=\frac{c\left(1-\psi \xi \right)\left(\beta_{2}H_{A}+\beta_{3}A_{A}+H_{U}\beta_{1}\right)S}{{\left(S+H_{U}+H_{A}+H_{T}+A_{A}+A_{T}\right)}^{2}}-\frac{c\left(1-\psi \xi \right)\beta_{2}S}{S+H_{U}+H_{A}+H_{T}+A_{A}+A_{T}},$$

$$g_{6}=\frac{c\left(1-\psi \xi \right)\beta_{2}S}{S+H_{U}+H_{A}+H_{T}+A_{A}+A_{T}}-\frac{c\left(1-\psi \xi \right)\left(\beta_{2}H_{A}+\beta_{3}A_{A}+H_{U}\beta_{1}\right)S}{{\left(S+H_{U}+H_{A}+H_{T}+A_{A}+A_{T}\right)}^{2}}$$

$$g_{7}=\frac{c\left(1-\psi \xi \right)\left(\beta_{2}H_{A}+\beta_{3}A_{A}+H_{U}\beta_{1}\right)S}{{\left(S+H_{U}+H_{A}+H_{T}+A_{A}+A_{T}\right)}^{2}}-\frac{c\left(1-\psi \xi \right)\beta_{3}S}{S+H_{U}+H_{A}+H_{T}+A_{A}+A_{T}},$$

$$g_{8}=\frac{c\left(1-\psi \xi \right)\beta_{3}S}{S+H_{U}+H_{A}+H_{T}+A_{A}+A_{T}}-\frac{c\left(1-\psi \xi \right)\left(\beta_{2}H_{A}+\beta_{3}A_{A}+H_{U}\beta_{1}\right)S}{{\left(S+H_{U}+H_{A}+H_{T}+A_{A}+A_{T}\right)}^{2}}$$

The resulting Jacobian matrix of (14) at *E*_0_ is

$$|A-\mathrm{\lambda I}|=\left|\begin{array}{cccccc} -\mu -\lambda & -\Lambda \zeta -c\left(1-\psi \xi \right)\beta_{1} & -c\left(1-\psi \xi \right)\beta_{2} & 0 & -c\left(1-\psi \xi \right)\beta_{3} & 0\\ 0 & \Lambda \zeta +c\left(1-\psi \xi \right)\beta_{1}-\alpha -\rho -\mu -\lambda & c\left(1-\psi \xi \right)\beta_{2} & 0 & c\left(1-\psi \xi \right)\beta_{3} & 0\\ 0 & \alpha & -\theta_{1}-\mu -\lambda & \upsilon_{1} & 0 & 0\\ 0 & 0 & \theta_{1} & -\upsilon_{1}-\mu -\lambda & 0 & 0\\ 0 & \rho & 0 & 0 & -\theta_{2}-d_{a}-\mu -\lambda & \upsilon_{2}\\ 0 & 0 & 0 & 0 & \theta_{2} & -\upsilon_{2}-d_{a}-\mu -\lambda \end{array}\right|$$ (16)

from (15) the first three eigenvalues are given as $\lambda_{1}=-\mu ,\lambda_{2}=-\left(v_{1}+\mu \right),\lambda_{3}=-\left(\theta_{1}+\mu \right)$ and the roots of the resulting quadratic equation is obtained as:

$$\begin{array}{l} f\left(\lambda \right)=\lambda^{3}+\left(c{\psi \xi \beta }_{1}-\zeta \Lambda -c\beta_{1}+M_{1}+M_{2}+M_{3}\right)\lambda^{2}+(\beta_{3}\text{cψρξ}+\mathrm{c\psi \xi}M_{2}\beta_{1}+\mathrm{c\psi \xi}M_{3}\beta_{1}-\Lambda \zeta M_{2}-\Lambda \zeta M_{3}-\beta_{3}\mathrm{c\rho}\\ \;-{\mathrm{cM}}_{2}\beta_{1}-{\mathrm{cM}}_{3}\beta_{1}+M_{1}M_{2}+M_{1}M_{3}+M_{2}M_{3}-\theta_{2}\upsilon_{2})\lambda +\beta_{3}\text{cψρξ}M_{3}+\mathrm{c\psi \xi}M_{2}M_{3}\beta_{1}-c{\psi \xi \beta }_{1}\theta_{2}\upsilon_{2}\\ \;-\Lambda \zeta M_{2}M_{3}+\Lambda {\zeta \theta }_{2}\upsilon_{2}-\beta_{3}\mathrm{c\rho}M_{3}-{\mathrm{cM}}_{2}M_{3}\beta_{1}+c\beta_{1}\theta_{2}\upsilon_{2}+M_{1}M_{2}M_{3}-M_{1}\theta_{2}\upsilon_{2} \end{array}$$ (17)

Because all parameters of the model are assumed to be positive, *λ*_4_ < 0, *λ*_5_ < 0, *λ*_6_ < 0. Evidently, if *R*_0_ < 1, the roots of *f*( *λ*) have negative real parts, implying that *E*_0_ is locally asymptotically stable (LAS) when *R*_0_ < 1; if *R*_0_ > 1, the roots of *f*( *λ*) are real and some are positive, implying that *E*_0_ is unstable.

**Theorem 3:** If *R*_0_ < 1, the disease free equilibrium is asymptotically stable globally for system (1).

**Proof:** The comparison theorem, as demonstrated by Ref. 55 proves the global stability of the disease-free equilibrium. We rename the infected class: $\frac{\mathrm{dx}}{\mathrm{dt}}=\left(F-V\right)X-\mathrm{JX},X=\left(H_{U},H_{A},H_{T},A_{A},A_{T}\right)$ where,

$$F=\left(\begin{array}{ccccc} c\left(1-\psi \xi \right)\beta_{1} & c\left(1-\psi \xi \right)\beta_{2} & 0 & c\left(1-\psi \xi \right)\beta_{3} & 0\\ 0 & 0 & 0 & 0 & 0\\ 0 & 0 & 0 & 0 & 0\\ 0 & 0 & 0 & 0 & 0\\ 0 & 0 & 0 & 0 & 0 \end{array}\right),V=\left(\begin{array}{ccccc} M_{1}-\Lambda \zeta & 0 & 0 & 0 & 0\\ -\alpha & M_{2} & -\upsilon_{1} & 0 & 0\\ 0 & -\theta_{1} & M_{3} & 0 & 0\\ -\rho & 0 & 0 & M_{4} & -\upsilon_{2}\\ 0 & 0 & 0 & -\theta_{2} & M_{5} \end{array}\right)$$ (18)

Then all of the matrix *F* − *V* eigenvalues have negative real parts, i.e So that

$$J=\left(1-\frac{S}{N}\right)\left|\begin{array}{ccccc} c\left(1-\psi \xi \right)\beta_{1}+\Lambda \zeta -M_{1}-\lambda & c\left(1-\psi \xi \right)\beta_{2} & 0 & c\left(1-\psi \xi \right)\beta_{3} & 0\\ \alpha & -M_{2}-\lambda & \upsilon_{1} & 0 & 0\\ 0 & \theta_{1} & -M_{3}-\lambda & 0 & 0\\ \rho & 0 & 0 & -M_{4}-\lambda & \upsilon_{2}\\ 0 & 0 & 0 & \theta_{2} & -M_{5}-\lambda \end{array}\right|=0$$ (19)

$$\begin{array}{l} \lambda^{5}-\left(c\beta_{1}-c{\psi \xi \beta }_{1}+\Lambda \zeta -M_{1}-M_{2}-M_{3}-M_{4}-M_{5}\right)\lambda^{4}-(\Lambda \zeta M_{2}+\Lambda \zeta M_{3}+\Lambda \zeta M_{4}+\Lambda \zeta M_{5}-\mathrm{\xi c}{\psi \beta }_{2}\alpha -c{\text{ψρξβ}}_{3}\\ \;-\mathrm{c\psi \xi}M_{2}\beta_{1}-\mathrm{c\psi \xi}M_{3}\beta_{1}-\mathrm{c\psi \xi}M_{4}\beta_{1}-\mathrm{c\psi \xi}M_{5}\beta_{1}+c\beta_{2}\alpha +\mathrm{\rho c}\beta_{3}+{\mathrm{cM}}_{2}\beta_{1}+{\mathrm{cM}}_{3}\beta_{1}+{\mathrm{cM}}_{4}\beta_{1}+{\mathrm{cM}}_{5}\beta_{1}-M_{2}M_{1}-M_{3}M_{1}\\ \;-M_{4}M_{1}-M_{1}M_{5}-M_{3}M_{2}-M_{2}M_{4}-M_{2}M_{5}-M_{3}M_{4}-M_{3}M_{5}-M_{4}M_{5}+\upsilon_{1}\theta_{1}+\theta_{2}\upsilon_{2})\lambda^{3}-(\Lambda \zeta M_{2}M_{3}+\Lambda \zeta M_{2}M_{4}\\ \;+\Lambda \zeta M_{2}M_{5}+\Lambda \zeta M_{3}M_{4}+\Lambda \zeta M_{3}M_{5}+\Lambda \zeta M_{4}M_{5}-\text{αcψξ}M_{3}\beta_{2}-\text{αcψξ}M_{4}\beta_{2}-\text{αcψξ}M_{5}\beta_{2}-\text{cψρξ}M_{2}\beta_{3}-\text{cψρξ}M_{3}\beta_{3}\\ \;-\text{cψρξ}M_{5}\beta_{3}-\mathrm{c\psi \xi}M_{2}M_{3}\beta_{1}-\mathrm{c\psi \xi}M_{2}M_{4}\beta_{1}-\mathrm{c\psi \xi}M_{2}M_{5}\beta_{1}-\mathrm{c\psi \xi}M_{3}M_{4}\beta_{1}-\mathrm{c\psi \xi}M_{3}M_{5}\beta_{1}-\mathrm{c\psi \xi}M_{4}M_{5}\beta_{1}+c{\psi \xi \beta }_{1}\theta_{1}\upsilon_{1}\\ \;+c{\psi \xi \beta }_{1}\theta_{2}\upsilon_{2}-\Lambda {\zeta \theta }_{1}\upsilon_{1}-\Lambda {\zeta \theta }_{2}\upsilon_{2}+\alpha {\mathrm{cM}}_{3}\beta_{2}+\alpha {\mathrm{cM}}_{4}\beta_{2}+\alpha {\mathrm{cM}}_{5}\beta_{2}+\mathrm{c\rho}M_{2}\beta_{3}+\mathrm{c\rho}M_{3}\beta_{3}+\mathrm{c\rho}M_{5}\beta_{3}+{\mathrm{cM}}_{2}M_{3}\beta_{1}\\ \;+{\mathrm{cM}}_{2}M_{4}\beta_{1}+{\mathrm{cM}}_{2}M_{5}\beta_{1}+{\mathrm{cM}}_{3}M_{4}\beta_{1}+{\mathrm{cM}}_{3}M_{5}\beta_{1}+{\mathrm{cM}}_{4}M_{5}\beta_{1}-c\beta_{1}\theta_{1}\upsilon_{1}-c\beta_{1}\theta_{2}\upsilon_{2}-M_{1}M_{2}M_{3}-M_{1}M_{2}M_{4}-M_{1}M_{2}M_{5}\\ \;-M_{1}M_{3}M_{4}-M_{1}M_{3}M_{5}-M_{1}M_{4}M_{5}+M_{1}\theta_{1}\upsilon_{1}+M_{1}\theta_{2}\upsilon_{2}-M_{2}M_{3}M_{4}-M_{2}M_{3}M_{5}-M_{2}M_{4}M_{5}+M_{2}\theta_{2}\upsilon_{2}-M_{3}M_{4}M_{5}\\ \;+M_{3}\theta_{2}\upsilon_{2}+M_{4}\theta_{1}\upsilon_{1}+M_{5}\theta_{1}\upsilon_{1})\lambda^{2}-(\mathrm{\alpha c}{\psi \xi \beta }_{2}\theta_{2}\upsilon_{2}-\text{αcψξ}M_{3}M_{4}\beta_{2}-\text{αcψξ}M_{3}M_{5}\beta_{2}-\text{αcψξ}M_{4}M_{5}\beta_{2}-\text{cψρξ}M_{2}M_{3}\beta_{3}\\ \;-\text{cψρξ}M_{2}M_{5}\beta_{3}-\text{cψρξ}M_{3}M_{5}\beta_{3}+c{\text{ψρξβ}}_{3}\theta_{1}\upsilon_{1}-\mathrm{c\psi \xi}M_{2}M_{3}M_{4}\beta_{1}-\mathrm{c\psi \xi}M_{2}M_{3}M_{5}\beta_{1}-\mathrm{c\psi \xi}M_{2}M_{4}M_{5}\beta_{1}+\mathrm{c\psi \xi}M_{2}\beta_{1}\theta_{2}\upsilon_{2}\\ \;-\mathrm{c\psi \xi}M_{3}M_{4}M_{5}\beta_{1}+\mathrm{c\psi \xi}M_{3}\beta_{1}\theta_{2}\upsilon_{2}+\mathrm{c\psi \xi}M_{4}\beta_{1}\theta_{1}\upsilon_{1}+\mathrm{c\psi \xi}M_{5}\beta_{1}\theta_{1}\upsilon_{1}+\Lambda \zeta M_{2}M_{3}M_{4}+\Lambda \zeta M_{2}M_{3}M_{5}+\Lambda \zeta M_{2}M_{4}M_{5}\\ \;-\Lambda \zeta M_{2}\theta_{2}\upsilon_{2}+\Lambda \zeta M_{3}M_{4}M_{5}-\Lambda \zeta M_{3}\theta_{2}\upsilon_{2}-\Lambda \zeta M_{4}\theta_{1}\upsilon_{1}-\Lambda \zeta M_{5}\theta_{1}\upsilon_{1}+\alpha {\mathrm{cM}}_{3}M_{4}\beta_{2}+\alpha {\mathrm{cM}}_{3}M_{5}\beta_{2}+\alpha {\mathrm{cM}}_{4}M_{5}\beta_{2}\\ \;-\mathrm{\alpha c}\beta_{2}\theta_{2}\upsilon_{2}+\mathrm{c\rho}M_{2}M_{3}\beta_{3}+\mathrm{c\rho}M_{2}M_{5}\beta_{3}+\mathrm{c\rho}M_{3}M_{5}\beta_{3}-c{\rho \beta }_{3}\theta_{1}\upsilon_{1}+{\mathrm{cM}}_{2}M_{3}M_{4}\beta_{1}+{\mathrm{cM}}_{2}M_{3}M_{5}\beta_{1}+{\mathrm{cM}}_{2}M_{4}M_{5}\beta_{1}\\ \;-{\mathrm{cM}}_{2}\beta_{1}\theta_{2}\upsilon_{2}+{\mathrm{cM}}_{3}M_{4}M_{5}\beta_{1}-{\mathrm{cM}}_{3}\beta_{1}\theta_{2}\upsilon_{2}-{\mathrm{cM}}_{4}\beta_{1}\theta_{1}\upsilon_{1}-{\mathrm{cM}}_{5}\beta_{1}\theta_{1}\upsilon_{1}-M_{1}M_{2}M_{3}M_{4}-M_{1}M_{2}M_{3}M_{5}-M_{1}M_{2}M_{4}M_{5}\\ \;+M_{1}M_{2}\theta_{2}\upsilon_{2}-M_{1}M_{3}M_{4}M_{5}+M_{1}M_{3}\theta_{2}\upsilon_{2}+M_{1}M_{4}\theta_{1}\upsilon_{1}+M_{1}M_{5}\theta_{1}\upsilon_{1}-M_{2}M_{3}M_{4}M_{5}+M_{2}M_{3}\theta_{2}\upsilon_{2}+M_{4}M_{5}\theta_{1}\upsilon_{1}\\ \;-\theta_{1}\theta_{2}\upsilon_{1}\upsilon_{2})\lambda +\text{αcψξ}M_{3}M_{4}M_{5}\beta_{2}-\text{αcψξ}M_{3}\beta_{2}\theta_{2}\upsilon_{2}+\text{cψρξ}M_{2}M_{3}M_{5}\beta_{3}-\text{cψρξ}M_{5}\beta_{3}\theta_{1}\upsilon_{1}+\mathrm{c\psi \xi}M_{2}M_{3}M_{4}M_{5}\beta_{1}\\ \;-\mathrm{c\psi \xi}M_{2}M_{3}\beta_{1}\theta_{2}\upsilon_{2}-\mathrm{c\psi \xi}M_{4}M_{5}\beta_{1}\theta_{1}\upsilon_{1}+c{\psi \xi \beta }_{1}\theta_{1}\theta_{2}\upsilon_{1}\upsilon_{2}-\Lambda \zeta M_{2}M_{3}M_{4}M_{5}+\Lambda \zeta M_{2}M_{3}\theta_{2}\upsilon_{2}+\Lambda \zeta M_{4}M_{5}\theta_{1}\upsilon_{1}\\ \;-\Lambda {\zeta \theta }_{1}\theta_{2}\upsilon_{1}\upsilon_{2}-\alpha {\mathrm{cM}}_{3}M_{4}M_{5}\beta_{2}+\alpha {\mathrm{cM}}_{3}\beta_{2}\theta_{2}\upsilon_{2}-\mathrm{c\rho}M_{2}M_{3}M_{5}\beta_{3}+\mathrm{c\rho}M_{5}\beta_{3}\theta_{1}\upsilon_{1}-{\mathrm{cM}}_{2}M_{3}M_{4}M_{5}\beta_{1}\\ \;+{\mathrm{cM}}_{2}M_{3}\beta_{1}\theta_{2}\upsilon_{2}+{\mathrm{cM}}_{4}M_{5}\beta_{1}\theta_{1}\upsilon_{1}-c\beta_{1}\theta_{1}\theta_{2}\upsilon_{1}\upsilon_{2}+M_{1}M_{2}M_{3}M_{4}M_{5}-M_{1}M_{2}M_{3}\theta_{2}\upsilon_{2}-M_{1}M_{4}M_{5}\theta_{1}\upsilon_{1}+M_{1}\theta_{1}\theta_{2}\upsilon_{1}\upsilon_{2} \end{array}$$ (20)

Equation (20) has four (4) negative roots by Descartes rule of signs if

$$\begin{array}{l} ({\text{αcψξM}}_{3}M_{4}M_{5}\beta_{2}-{\text{αcψξM}}_{3}\beta_{2}\theta_{2}\upsilon_{2}+{\text{cψρξM}}_{2}M_{3}M_{5}\beta_{3}-{\text{cψρξM}}_{5}\beta_{3}\theta_{1}\upsilon_{1}+{\text{cψξM}}_{2}M_{3}M_{4}M_{5}\beta_{1}-{\text{cψξM}}_{2}M_{3}\beta_{1}\theta_{2}\upsilon_{2}\\ \;-{\text{cψξM}}_{4}M_{5}\beta_{1}\theta_{1}\upsilon_{1}+{\text{cψξβ}}_{1}\theta_{1}\theta_{2}\upsilon_{1}\upsilon_{2}-\Lambda {\mathrm{\zeta M}}_{2}M_{3}M_{4}M_{5}+\Lambda {\mathrm{\zeta M}}_{2}M_{3}\theta_{2}\upsilon_{2}+\Lambda {\mathrm{\zeta M}}_{4}M_{5}\theta_{1}\upsilon_{1}-\Lambda {\zeta \theta }_{1}\theta_{2}\upsilon_{1}\upsilon_{2}-{\mathrm{\alpha cM}}_{3}M_{4}M_{5}\beta_{2}\\ \;+{\mathrm{\alpha cM}}_{3}\beta_{2}\theta_{2}\upsilon_{2}-{\mathrm{c\rho M}}_{2}M_{3}M_{5}\beta_{3}+{\mathrm{c\rho M}}_{5}\beta_{3}\theta_{1}\upsilon_{1}-{\mathrm{cM}}_{2}M_{3}M_{4}M_{5}\beta_{1}+{\mathrm{cM}}_{2}M_{3}\beta_{1}\theta_{2}\upsilon_{2}+{\mathrm{cM}}_{4}M_{5}\beta_{1}\theta_{1}\upsilon_{1}-{\mathrm{c\beta}}_{1}\theta_{1}\theta_{2}\upsilon_{1}\upsilon_{2}\\ \;+M_{1}M_{2}M_{3}M_{4}M_{5}-M_{1}M_{2}M_{3}\theta_{2}\upsilon_{2}-M_{1}M_{4}M_{5}\theta_{1}\upsilon_{1}+M_{1}\theta_{1}\theta_{2}\upsilon_{1}\upsilon_{2})<[({\mathrm{c\beta}}_{1}-{\text{cψξβ}}_{1}+\Lambda \zeta -M_{1}-M_{2}-M_{3}-M_{4}-M_{5})\\ \;\times (\Lambda {\mathrm{\zeta M}}_{2}+\Lambda {\mathrm{\zeta M}}_{3}+\Lambda {\mathrm{\zeta M}}_{4}+\Lambda {\mathrm{\zeta M}}_{5}-{\text{ξcψβ}}_{2}\alpha -{\text{cψρξβ}}_{3}-{\text{cψξM}}_{2}\beta_{1}-{\text{cψξM}}_{3}\beta_{1}-{\text{cψξM}}_{4}\beta_{1}-{\text{cψξM}}_{5}\beta_{1}+{\mathrm{c\beta}}_{2}\alpha +{\mathrm{\rho c\beta}}_{3}\\ \;+{\mathrm{cM}}_{2}\beta_{1}+{\mathrm{cM}}_{3}\beta_{1}+{\mathrm{cM}}_{4}\beta_{1}+{\mathrm{cM}}_{5}\beta_{1}-M_{2}M_{1}-M_{3}M_{1}-M_{4}M_{1}-M_{1}M_{5}-M_{3}M_{2}-M_{2}M_{4}-M_{2}M_{5}-M_{3}M_{4}-M_{3}M_{5}-M_{4}M_{5}\\ \;+\upsilon_{1}\theta_{1}+\theta_{2}\upsilon_{2})(\Lambda {\mathrm{\zeta M}}_{2}M_{3}+\Lambda {\mathrm{\zeta M}}_{2}M_{4}+\Lambda {\mathrm{\zeta M}}_{2}M_{5}+\Lambda {\mathrm{\zeta M}}_{3}M_{4}+\Lambda {\mathrm{\zeta M}}_{3}M_{5}+\Lambda {\mathrm{\zeta M}}_{4}M_{5}-{\text{αcψξM}}_{3}\beta_{2}-{\text{αcψξM}}_{4}\beta_{2}\\ \;-{\text{αcψξM}}_{5}\beta_{2}-{\text{cψρξM}}_{2}\beta_{3}-{\text{cψρξM}}_{3}\beta_{3}-{\text{cψρξM}}_{5}\beta_{3}-{\text{cψξM}}_{2}M_{3}\beta_{1}-{\text{cψξM}}_{2}M_{4}\beta_{1}-{\text{cψξM}}_{2}M_{5}\beta_{1}-{\text{cψξM}}_{3}M_{4}\beta_{1}-{\text{cψξM}}_{3}M_{5}\beta_{1}\\ \;-{\text{cψξM}}_{4}M_{5}\beta_{1}+{\text{cψξβ}}_{1}\theta_{1}\upsilon_{1}+{\text{cψξβ}}_{1}\theta_{2}\upsilon_{2}-\Lambda {\zeta \theta }_{1}\upsilon_{1}-\Lambda {\zeta \theta }_{2}\upsilon_{2}+{\mathrm{\alpha cM}}_{3}\beta_{2}+{\mathrm{\alpha cM}}_{4}\beta_{2}+{\mathrm{\alpha cM}}_{5}\beta_{2}+{\mathrm{c\rho M}}_{2}\beta_{3}+{\mathrm{c\rho M}}_{3}\beta_{3}\\ \;+{\mathrm{c\rho M}}_{5}\beta_{3}+{\mathrm{cM}}_{2}M_{3}\beta_{1}+{\mathrm{cM}}_{2}M_{4}\beta_{1}+{\mathrm{cM}}_{2}M_{5}\beta_{1}+{\mathrm{cM}}_{3}M_{4}\beta_{1}+{\mathrm{cM}}_{3}M_{5}\beta_{1}+{\mathrm{cM}}_{4}M_{5}\beta_{1}-{\mathrm{c\beta}}_{1}\theta_{1}\upsilon_{1}-{\mathrm{c\beta}}_{1}\theta_{2}\upsilon_{2}-M_{1}M_{2}M_{3}\\ \;-M_{1}M_{2}M_{4}-M_{1}M_{2}M_{5}-M_{1}M_{3}M_{4}-M_{1}M_{3}M_{5}-M_{1}M_{4}M_{5}+M_{1}\theta_{1}\upsilon_{1}+M_{1}\theta_{2}\upsilon_{2}-M_{2}M_{3}M_{4}-M_{2}M_{3}M_{5}-M_{2}M_{4}M_{5}\\ \;+M_{2}\theta_{2}\upsilon_{2}-M_{3}M_{4}M_{5}+M_{3}\theta_{2}\upsilon_{2}+M_{4}\theta_{1}\upsilon_{1}+M_{5}\theta_{1}\upsilon_{1})({\text{αcψξβ}}_{2}\theta_{2}\upsilon_{2}-{\text{αcψξM}}_{3}M_{4}\beta_{2}-{\text{αcψξM}}_{3}M_{5}\beta_{2}-{\text{αcψξM}}_{4}M_{5}\beta_{2}\\ \;-{\text{cψρξM}}_{2}M_{3}\beta_{3}-{\text{cψρξM}}_{2}M_{5}\beta_{3}-{\text{cψρξM}}_{3}M_{5}\beta_{3}+{\text{cψρξβ}}_{3}\theta_{1}\upsilon_{1}-{\text{cψξM}}_{2}M_{3}M_{4}\beta_{1}-{\text{cψξM}}_{2}M_{3}M_{5}\beta_{1}-{\text{cψξM}}_{2}M_{4}M_{5}\beta_{1}\\ \;+{\text{cψξM}}_{2}\beta_{1}\theta_{2}\upsilon_{2}-{\text{cψξM}}_{3}M_{4}M_{5}\beta_{1}+{\text{cψξM}}_{3}\beta_{1}\theta_{2}\upsilon_{2}+{\text{cψξM}}_{4}\beta_{1}\theta_{1}\upsilon_{1}+{\text{cψξM}}_{5}\beta_{1}\theta_{1}\upsilon_{1}+\Lambda {\mathrm{\zeta M}}_{2}M_{3}M_{4}+\Lambda {\mathrm{\zeta M}}_{2}M_{3}M_{5}\\ \;+\Lambda {\mathrm{\zeta M}}_{2}M_{4}M_{5}-\Lambda {\mathrm{\zeta M}}_{2}\theta_{2}\upsilon_{2}+\Lambda {\mathrm{\zeta M}}_{3}M_{4}M_{5}-\Lambda {\mathrm{\zeta M}}_{3}\theta_{2}\upsilon_{2}-\Lambda {\mathrm{\zeta M}}_{4}\theta_{1}\upsilon_{1}-\Lambda {\mathrm{\zeta M}}_{5}\theta_{1}\upsilon_{1}+{\mathrm{\alpha cM}}_{3}M_{4}\beta_{2}+{\mathrm{\alpha cM}}_{3}M_{5}\beta_{2}+{\mathrm{\alpha cM}}_{4}M_{5}\beta_{2}\\ \;-{\mathrm{\alpha c\beta}}_{2}\theta_{2}\upsilon_{2}+{\mathrm{c\rho M}}_{2}M_{3}\beta_{3}+{\mathrm{c\rho M}}_{2}M_{5}\beta_{3}+{\mathrm{c\rho M}}_{3}M_{5}\beta_{3}-{\mathrm{c\rho \beta}}_{3}\theta_{1}\upsilon_{1}+{\mathrm{cM}}_{2}M_{3}M_{4}\beta_{1}+{\mathrm{cM}}_{2}M_{3}M_{5}\beta_{1}+{\mathrm{cM}}_{2}M_{4}M_{5}\beta_{1}\\ \;-{\mathrm{cM}}_{2}\beta_{1}\theta_{2}\upsilon_{2}+{\mathrm{cM}}_{3}M_{4}M_{5}\beta_{1}-{\mathrm{cM}}_{3}\beta_{1}\theta_{2}\upsilon_{2}-{\mathrm{cM}}_{4}\beta_{1}\theta_{1}\upsilon_{1}-{\mathrm{cM}}_{5}\beta_{1}\theta_{1}\upsilon_{1}-M_{1}M_{2}M_{3}M_{4}-M_{1}M_{2}M_{3}M_{5}-M_{1}M_{2}M_{4}M_{5}\\ \;+M_{1}M_{2}\theta_{2}\upsilon_{2}-M_{1}M_{3}M_{4}M_{5}+M_{1}M_{3}\theta_{2}\upsilon_{2}+M_{1}M_{4}\theta_{1}\upsilon_{1}+M_{1}M_{5}\theta_{1}\upsilon_{1}-M_{2}M_{3}M_{4}M_{5}+M_{2}M_{3}\theta_{2}\upsilon_{2}+M_{4}M_{5}\theta_{1}\upsilon_{1}-\theta_{1}\theta_{2}\upsilon_{1}\upsilon_{2})] \end{array}$$

Since $S\left(t\right)\leq \frac{\Lambda }{\mu }$ in the invariant set, *J* is a non-negative matrix. Hence, it follows that

$$\frac{\mathrm{dx}}{\mathrm{dt}}\leq \left(F-V\right)X$$

When *R*_0_ < 1, the eigenvalues of the matrix *F* − *V* are negative. As a result, the linearized differential equation is stable whenever *R*_0_ < 1 is positive. Since $\left(H_{U},H_{A},H_{T},A_{A},A_{T}\right)\to \left(0,0,0,0,0\right)$ as t→∞ . According to the comparison theorem, $\left(H_{U},H_{A},H_{T},A_{A},A_{T}\right)\to \left(0,0,0,0,0\right)$ as t→∞ . Substituting *H _U_* = *H _A_* = *H _T_* = *A _A_* = *A _T_* = 0 in (1) gives $S\left(t\right)\to S_{0}$ as t→∞ . Thus, $\left(S,H_{U},H_{A},H_{T},A_{A},A_{T}\right)\to \left(S_{0},0,0,0,0,0\right)$ as t→∞ for *R*_0_ < 1. Thus, *E*_0_ is globally asymptotically stable if *R*_0_ < 1.

### 4.2 The endemic equilibrium’s local and global stability; *E* ^*^

**Theorem 4:** The endemic steady state $E^{*}\left(S^{*},{H_{u}}^{*},H_{A}^{*},H_{T}^{*},A_{A}^{*},A_{T}^{*}\right)$ of the model is locally asymptotically stable (LAS) If *R*_0_ > 1.

**Proof:** We must now demonstrate the local stability of the endemic steady state. Assume *R*0 > 1.

The Jacobian matrix for the variables of system (1) is computed in the proof of Theorem 2 as in (14).

Hence, for the endemic equilibrium $\left(S^{*},H_{U}^{*},H_{A}^{*},H_{T}^{*},A_{A}^{*},A_{T}^{*})\right.$ , the Jacobian matrix and the determinantal equation at the endemic equilibrium is given as matrix in (15)

Clearly, the equation reduces to:

$$\left(-\theta_{1}-\mu -\lambda \right)\left(-v_{1}-\mu -\lambda \right)\left(-v_{2}-d_{a}-\mu -\lambda \right)\left(-\theta_{2}-d_{a}-\mu -\lambda \right)\left|\begin{array}{ll} g_{1}-\mu -\lambda & -\Lambda \zeta +g_{2}\\ g_{3} & \Lambda \zeta -\alpha +g_{4}-\mu -\rho -\lambda \end{array}\right|=0$$ (21)

The first four eigenvalues of (21) are given as:

$$\lambda_{1}=-\left(\theta_{1}+\mu \right),\lambda_{2}=-\left(v_{1}+\mu \right),\lambda_{3}=-\left(v_{2}+d_{a}+\mu \right),\lambda_{4}=-\left(\theta_{2}+d_{a}+\mu \right)$$

The eigenvalue of the remaining 2 × 2 is obtained from the characteristics equation below:

$$\lambda^{2}+\left(+\alpha -\Lambda \zeta -g_{1}-g4+2\mu +\rho \right)\lambda +\Lambda \zeta g_{1}+\Lambda \zeta g_{3}-\Lambda \zeta \mu -\alpha g_{1}+\alpha \mu +g_{1}g_{4}-g_{1}\mu -g_{1}\rho -g_{2}g_{3}-g_{4}\mu +\mu^{2}+\mu \rho$$ (22)

The determinants of the characteristic polynomial from (22) yield the following result:

$$f(\lambda )=\lambda_{2}+a_{1}\lambda +a_{0}.$$

Polynomials of order 2 satisfy the Routh-Hurwitz criterion, We know that *f*( *λ*) = 0 using Routh-Hurwitz criterion polynomials of order 2 is stable if and only if both coefficients in (22) satisfy the following conditions: *a _i_* > 0 From Eq. (22) the condition is satisfied. Therefore, EE is locally asymptotically stable.

**Theorem 5:** when R _0_<1, the equations of the model have a positive distinct endemic equilibrium, which is said to be globally asymptotically stable.

**Proof:** Considering the Lyapunov function, which is defined as

$$L\left(S^{*},H_{U}^{*},{H_{A}}^{*},H_{T}^{*},A_{A}^{*},A_{T}^{*}\right)=\left(S-S^{*}\ln\left(\frac{S}{S^{*}}\right)\right)+\left(H_{U}-H_{U}^{*}\ln\left(\frac{H_{U}}{H_{U}^{*}}\right)\right)+\left(H_{A}-H_{A}^{*}\ln\left(\frac{H_{A}}{H_{A}^{*}}\right)\right)+\left(H_{T}-H_{T}^{*}\ln\left(\frac{H_{T}}{H_{T}^{*}}\right)\right)+\left(A_{A}-A_{A}^{*}\ln\left(\frac{A_{A}}{A_{A}^{*}}\right)\right)+\left(A_{T}-A_{T}^{*}\ln\left(\frac{A_{T}}{A_{T}^{*}}\right)\right)$$

where *L* directly takes its derivative along the system as:

$$\frac{\mathrm{dL}}{\mathrm{dt}}=\left(1-\frac{S^{*}}{S}\right)\frac{\mathrm{dS}}{\mathrm{dt}}+\left(1-\frac{H_{U}^{*}}{H_{U}}\right)\frac{{\mathrm{dH}}_{U}}{\mathrm{dt}}+\left(1-\frac{H_{A}^{*}}{H_{A}}\right)\frac{{\mathrm{dH}}_{A}}{\mathrm{dt}}+\left(1-\frac{H_{T}^{*}}{H_{T}}\right)\frac{{\mathrm{dH}}_{T}}{\mathrm{dt}}+\left(1-\frac{A_{A}^{*}}{A_{A}}\right)\frac{{\mathrm{dA}}_{A}}{\mathrm{dt}}+\left(1-\frac{A_{T}^{*}}{A_{T}}\right)\frac{{\mathrm{dA}}_{T}}{\mathrm{dt}}$$

$$\begin{array}{l} \frac{\mathrm{dL}}{\mathrm{dt}}=\left(1-\frac{S^{*}}{S}\right)\langle \Lambda \left(1-\zeta H_{U}\right)-\left(\left(\frac{{\mathrm{cb}}_{h}\left(1-\psi \xi \right)\beta_{1}H_{U}+\beta_{2}H_{A}+\beta_{3}A_{A}}{N}\right)+\mu \right)S\rangle \\ \;+\left(1-\frac{H_{U}^{*}}{H_{U}}\right)\langle \left(\frac{{\mathrm{cb}}_{h}\left(1-\psi \xi \right)\beta_{1}H_{U}+\beta_{2}H_{A}+\beta_{3}A_{A}}{N}\right)S-(\alpha +\rho +\mu )H_{U}+\Lambda \zeta H_{U}\rangle \\ \;+\left(1-\frac{H_{A}^{*}}{H_{A}}\right)\langle \alpha H_{U}+\upsilon_{1}H_{T}-\left(\theta_{1}+\mu \right)H_{A}\rangle +\left(1-\frac{H_{T}^{*}}{H_{T}}\right)\langle \theta_{1}H_{A}-\left(\upsilon +\mu \right)H_{T}\rangle \\ \;+\left(1-\frac{A_{A}^{*}}{A_{A}}\right)\langle \rho H_{U}+\upsilon_{2}A_{T}-\left(\theta_{2}+d_{a}+\mu \right)A_{A}\rangle +\left(1-\frac{A_{T}^{*}}{A_{T}}\right)\langle \theta_{2}A_{A}-\left(\upsilon_{2}+d_{a}+\mu \right)A_{T}\rangle \end{array}$$

At equilibrium

$$\Lambda \left(1-\zeta H_{U}\right)={\mathrm{cb}}_{h}\left(1-\psi \xi \right)\left(\frac{\beta_{1}H_{U}^{*}+\beta_{2}H_{A}^{*}+\beta_{3}A_{A}^{*}}{N^{*}}\right)S^{*}+\mu S^{*}$$

$$\left(\alpha +\rho +\mu +\Lambda \zeta \right)={\mathrm{cb}}_{h}\left(1-\psi \xi \right)\left(\frac{\beta_{1}H_{U}^{*}+\beta_{2}H_{A}^{*}+\beta_{3}A_{A}^{*}}{{H_{U}}^{*}N^{*}}\right)S^{*}$$

$$\left(\theta_{1}+\mu \right)=\frac{\alpha H_{U}^{*}+\upsilon_{1}H_{T}^{*}}{H_{A}^{*}}$$

$$\left(\upsilon_{1}+\mu \right)=\frac{\theta_{1}H_{A}^{*}}{H_{T}^{*}}$$

$$\left(\theta_{2}+d_{a}+\mu \right)=\frac{\rho H_{U}^{*}}{A_{A}^{*}}+\frac{\upsilon_{2}A_{T}^{*}}{A_{A}^{*}}$$

$$\left(\upsilon_{2}+d_{a}+\mu \right)=\frac{\theta_{2}A_{A}^{*}}{A_{T}^{*}}$$

$$\begin{array}{l} \frac{\mathrm{dL}}{\mathrm{dt}}=\left(1-\frac{S^{*}}{S}\right)\langle \left(\frac{{\mathrm{cb}}_{h}\left(1-\psi \xi \right)\beta_{1}H_{U}^{*}+\beta_{2}H_{A}^{*}+\beta_{3}A_{A}^{*}}{N^{*}}\right)S^{*}+\mu S^{*}-\langle \left(\frac{{\mathrm{cb}}_{h}\left(1-\psi \xi \right)\beta_{1}H_{U}+\beta_{2}H_{A}+\beta_{3}A_{A}}{N}\right)+\mu \rangle S\rangle \\ \;+\left(1-\frac{H_{U}^{*}}{H_{U}}\right)\langle \left(\frac{{\mathrm{cb}}_{h}\left(1-\psi \xi \right)\beta_{1}H_{U}+\beta_{2}H_{A}+\beta_{3}A_{A}}{N}\right)S-\left(\frac{{\mathrm{cb}}_{h}\left(1-\psi \xi \right)\left(\beta_{1}H_{U}^{*}+\beta_{2}H_{A}^{*}+\beta_{3}A_{A}^{*}\right)}{H_{U}^{*}N^{*}}\right)S^{*}H_{U}\\ \;+\left(1-\frac{H_{A}^{*}}{H_{A}}\right)\langle \alpha H_{U}+\upsilon_{1}H_{T}-\left(\frac{\alpha H_{U}^{*}}{H_{A}^{*}}+\frac{\upsilon_{1}H_{T}^{*}}{H_{A}^{*}}\right)H_{A}\rangle +\left(1-\frac{H_{T}^{*}}{H_{T}}\right)\langle \theta_{1}H_{A}-\left(\frac{\theta_{1}H_{A}^{*}}{H_{T}}\right)H_{T}\rangle \\ \;+\left(1-\frac{A_{A}^{*}}{A_{A}}\right)\langle \rho H_{U}+\upsilon_{2}A_{T}-\left(\frac{\rho H_{U}^{*}}{A_{A}^{*}}+\frac{\upsilon_{2}A_{T}^{*}}{A_{A}^{*}}\right)A_{A}\rangle +\left(1-\frac{A_{T}^{*}}{A_{T}}\right)\langle \theta_{2}A_{A}-\frac{\theta_{2}A_{A}^{*}}{A_{T}^{*}}A_{T}\rangle \end{array}$$

$$\begin{array}{l} =\left(1-\frac{S^{*}}{S}\right)\langle \frac{{\mathrm{cb}}_{h}\left(1-\psi \xi \right)\beta_{1}H_{U}^{*}}{N^{*}}S^{*}+\frac{{\mathrm{cb}}_{h}\left(1-\psi \xi \right)\beta_{2}H_{A}^{*}}{N^{*}}S^{*}+\frac{{\mathrm{cb}}_{H}\left(1-\psi \xi \right)\beta_{3}A_{A}^{*}}{N^{*}}S^{*}+\mu S^{*}-\frac{{\mathrm{cb}}_{h}\left(1-\psi \xi \right)\beta_{1}H_{U}}{N}S\\ \;-\frac{{\mathrm{cb}}_{h}\left(1-\psi \xi \right)\beta_{2}H_{A}}{N}S-\frac{{\mathrm{cb}}_{h}\left(1-\psi \xi \right)\beta_{3}A_{A}}{N}S-\mathrm{\mu S}\rangle +\left(1-\frac{H_{U}^{*}}{H_{U}}\right)\langle \frac{{\mathrm{cb}}_{h}\left(1-\psi \xi \right)\beta_{1}H_{U}}{N}S+\frac{{\mathrm{cb}}_{h}\left(1-\psi \xi \right)\beta_{2}H_{A}}{N}S\\ \;+\frac{{\mathrm{cb}}_{h}\left(1-\psi \xi \right)\beta_{3}A_{A}}{N}S-\frac{{\mathrm{cb}}_{h}\left(1-\psi \xi \right)\beta_{1}H_{U}^{*}S^{*}H_{U}}{H_{U}^{*}N^{*}}-\frac{{\mathrm{cb}}_{h}\left(1-\psi \xi \right)\beta_{2}H_{A}^{*}S^{*}H_{U}}{H_{U}^{*}N^{*}}-\frac{{\mathrm{cb}}_{h}\left(1-\psi \xi \right)\beta_{3}A_{A}^{*}S^{*}H_{U}}{H_{U}^{*}N^{*}}S\rangle \\ \;+\left(1-\frac{H_{A}^{*}}{H_{A}}\right)\langle \alpha H_{U}+\upsilon_{1}H_{T}-\frac{\alpha H_{U}^{*}H_{A}}{H_{A}^{*}}-\frac{\upsilon_{1}H_{T}^{*}H_{A}}{H_{A}^{*}}\rangle +\left(1-\frac{H_{T}^{*}}{H_{T}}\right)\left(\theta_{1}H_{A}-\frac{\theta_{1}H_{A}^{*}H_{T}}{H_{T}}\right)\\ \;+\left(1-\frac{A_{A}^{*}}{A_{A}}\right)\langle \rho H_{U}+\upsilon_{2}A_{T}-\frac{\rho H_{U}^{*}A_{A}}{A_{A}^{*}}-\frac{\upsilon_{2}A_{T}^{*}A_{A}}{A_{A}^{*}}\rangle +\left(1-\frac{A_{T}^{*}}{A_{T}}\right)\left(\theta_{2}A_{A}-\frac{\theta_{2}A_{A}A_{T}}{A_{T}^{*}}\right) \end{array}$$

$$\begin{array}{l} =\left(1-\frac{S^{*}}{S}\right)\langle \frac{{\mathrm{cb}}_{h}\left(1-\psi \xi \right)\beta_{1}H_{U}^{*}S}{N}\left(1-\frac{H_{U}^{*}S^{*}N}{H_{U}{\mathrm{SN}}^{*}}\right)-{\mathrm{cb}}_{h}\left(1-\psi \xi \right)\beta_{2}H_{A}S\left(1-\frac{H_{A}^{*}S^{*}N}{H_{A}{\mathrm{SN}}^{*}}\right)\\ \;-{\mathrm{cb}}_{h}\left(1-\psi \xi \right)\beta_{3}A_{A}S\left(1-\frac{H_{A}^{*}S^{*}N}{A_{A}{\mathrm{SN}}^{*}}\right)-\mathrm{\mu S}(1-\frac{S^{*}}{S})+\left(1-\frac{H_{U}^{*}}{H_{U}}\right)\\ \;\langle \frac{{\mathrm{cb}}_{h}\left(1-\psi \xi \right)\beta_{1}H_{U}S}{N}\left(1-\frac{H_{U}^{*}S^{*}N}{H_{U}^{*}{\mathrm{SN}}^{*}}\right)+\frac{{\mathrm{cb}}_{h}\left(1-\psi \xi \right)\beta_{2}H_{A}S}{N}\left(1-\frac{H_{A}^{*}S^{*}H_{U}}{H_{A}{\mathrm{SH}}_{U}^{*}N^{*}}\right)+\frac{{\mathrm{cb}}_{h}\left(1-\psi \xi \right)\beta_{3}A_{A}S}{N}\left(1-\frac{A_{A}^{*}S^{*}H_{U}}{A_{A}{\mathrm{SH}}_{U}^{*}N^{*}}\right)\rangle \\ \;+(1-\frac{H_{A}^{*}}{H_{A}})\langle \alpha H_{U}\left(1-\frac{H_{U}^{*}}{H_{U}}\right)\left(1-\frac{H_{A}^{*}}{H_{A}}\right)+\upsilon H_{T}\left(1-\frac{H_{T}^{*}}{H_{T}}\right)\left(1-\frac{H_{A}^{*}}{H_{A}}\right)\rangle \\ \;+\left(1-\frac{H_{T}^{*}}{H_{T}}\right)\langle (\theta_{1}H_{A}\left(1-\frac{H_{A}^{*}}{H_{A}}\right)\left(1-\frac{H_{T}^{*}}{H_{T}}\right)\rangle +(1-\frac{A_{A}^{*}}{A_{A}})\langle \rho H_{U}\left(1-\frac{H_{U}^{*}}{H_{U}}\right)\left(1-\frac{A_{A}^{*}}{A_{A}}\right)+\upsilon_{2}A_{T}(1-\frac{A_{T}^{*}}{A_{T}}\left(1-\frac{A_{A}^{*}}{A_{A}}\right)\rangle \\ \;+\left(1-\frac{A_{T}^{*}}{A_{T}}\right)\langle \theta_{2}A_{A}\left(1-\frac{A_{A}^{*}}{A_{A}}\right)\left(1-\frac{A_{T}^{*}A_{T}}{A_{T}}\right)=-\mathrm{\mu S}{\left(1-\frac{S^{*}}{S}\right)}^{2}+P_{1}\left(S,H_{U},H_{A},H_{T},A_{A},A_{T}\right)+P_{2}\left(S,H_{U},H_{A},H_{T},A_{A},A_{T}\right) \end{array}$$

where

$$P_{1}\left(S,H_{U},H_{A},H_{T},A_{A},A_{T}\right)=-\frac{{\mathrm{cb}}_{h}\left(1-\psi \xi \right)\beta_{1}H_{U}S}{N}\left(1-\frac{S^{*}}{S}\right)\left(1-\frac{H_{U}^{*}S^{*}N}{H_{U}{\mathrm{SN}}^{*}}\right)-\frac{{\mathrm{cb}}_{h}\left(1-\psi \xi \right)\beta_{2}H_{A}^{*}S^{*}N}{H_{A}{\mathrm{SN}}^{*}}\left(1-\frac{S^{*}}{S}\right)\left(1-\frac{H_{A}^{*}S^{*}N}{H_{A}{\mathrm{SN}}^{*}}\right)-\frac{{\mathrm{cb}}_{h}\left(1-\psi \xi \right)\beta_{3}A_{A}S}{N}\left(1-\frac{S^{*}}{S}\right)\left(1-\frac{A_{A}^{*}S^{*}N}{A_{A}{\mathrm{SN}}^{*}}\right)$$

$$P_{2}\left(S,H_{U},H_{A},H_{T},A_{A},A_{T}\right)=\text{All others}$$

$$P_{1}\leq 0\;\text{whenever}\;H_{U}{\mathrm{SN}}^{*}\geq H_{U}^{*}S^{*}N,\;H_{A}{\mathrm{SN}}^{*}\geq H_{A}^{*}S^{*}N,A_{A}{\mathrm{SN}}^{*}\geq A_{A}^{*}S^{*}N$$ (23)

$$\begin{array}{l} P_{2}\leq 0\;\text{whenever}\;{H_{U}}^{*}{\mathrm{SN}}^{*}\geq H_{U}^{*}S^{*}N,\;H_{A}{\mathrm{SH}}_{U}^{*}N^{*}\geq H_{A}^{*}S^{*}H_{U},A_{A}{\mathrm{SH}}_{U}^{*}N^{*}\geq A_{A}^{*}S^{*}H_{U},H_{U}H_{A}^{*}\geq H_{U}^{*}H_{A},H_{T}^{*}H_{A},\\ H_{U}A_{A}^{*}\geq H_{U}^{*}A_{A},A_{A}A_{T}\geq A_{A}^{*}A_{T} \end{array}$$ (24)

Thus

$$\frac{\mathrm{dL}}{\mathrm{dt}}\leq 0$$

if (23) and (24) holds.

Hence, by Lasalle theorem, the equilibrium is globally asymptotically stable in the feasible region $R_{+}^{6}$ .

### 4.3 Sensitivity indices

Knowing the relative relevance of the different factors involved in HIV transmission and prevalence is vital for deciding how effectively to minimize human morbidity and mortality rate due to HIV infections. Sensitivity analysis is performed in this sub-section to assess the resilience of factors that have a strong impact on the basic reproduction number, *R*_0_, so that suitable intervention strategies may be implemented.

The effect of HIV testing and treatment on HIV/AIDS dynamics was studied using the elasticity of *ReH* with respect to *α and θ.* Using the method described in ^57^^,^^64^^,^^65^ to compute the elasticity ^58^ of *R _eH_* with respect to *α and θ* as shown in Equation (25)

$$\frac{\alpha \theta }{R_{\mathrm{eH}}}\frac{\partial R_{\mathrm{eH}}}{\partial \alpha \theta }=\frac{c\left(1-\psi \xi \right)}{\zeta \Lambda -M_{1}}+\frac{c\left(1-\psi \xi \right)\alpha M_{3}}{\left(M_{2}M_{3}-\theta_{1}\upsilon_{1}\right)\left(\zeta \Lambda -M_{1}\right)}+\frac{c\left(1-\psi \xi \right)\rho M_{4}}{\left(M_{4}^{2}-\theta_{2}\upsilon_{2}\right)\left(\zeta \Lambda -M_{1}\right)}$$ (25)

*Interpretation of the sensitivity indices*

Table 1’s sensitivity indices are read as follows: Positive indices indicate that the corresponding basic reproduction number increases (decreases) as those parameters increase (decrease). Negative indices, on the other hand, indicate that increasing (decreasing) those parameters reduces the associated basic reproduction number (increases).

**Table 1.** Sensitivity indices of *R*_0._

Parameter	Sensitivity index	Parameter	Sensitivity index
Λ	+	*α*	-
*ζ*	+	*μ*	-
*β*_1_	+	*ρ*	-
*β*_2_	+	*d _a_*	-
*β*_3_	+	*θ*_1_	-
*c*	+	*θ*_2_	-
*υ*_1_	+		
*υ*_2_	+		

The endemicity of HIV infection increases when the values of *β _i_*, *i* = 1, 2, 3, *υ*, and *c* are increased; when the values of alpha and mu are decreased, the endemicity of HIV infection decreases.

As a result, interventions should aim to reduce the annual average number of sexual partners acquired, *c*, the number of defaulters lost to follow-up, *υ*, and the likelihood of HIV transmission per sexual contact, *β _i_*, *i* = 1, 2, 3, because the rate of progression from HIV to AIDS is increasing, *ρ*, indicates rapid progression to AIDS. In addition, effective condom use should be mandated as a precautionary measure to reduce the rate of HIV/AIDS transmission.

## 5. Numerical simulation

**Figure 2.**
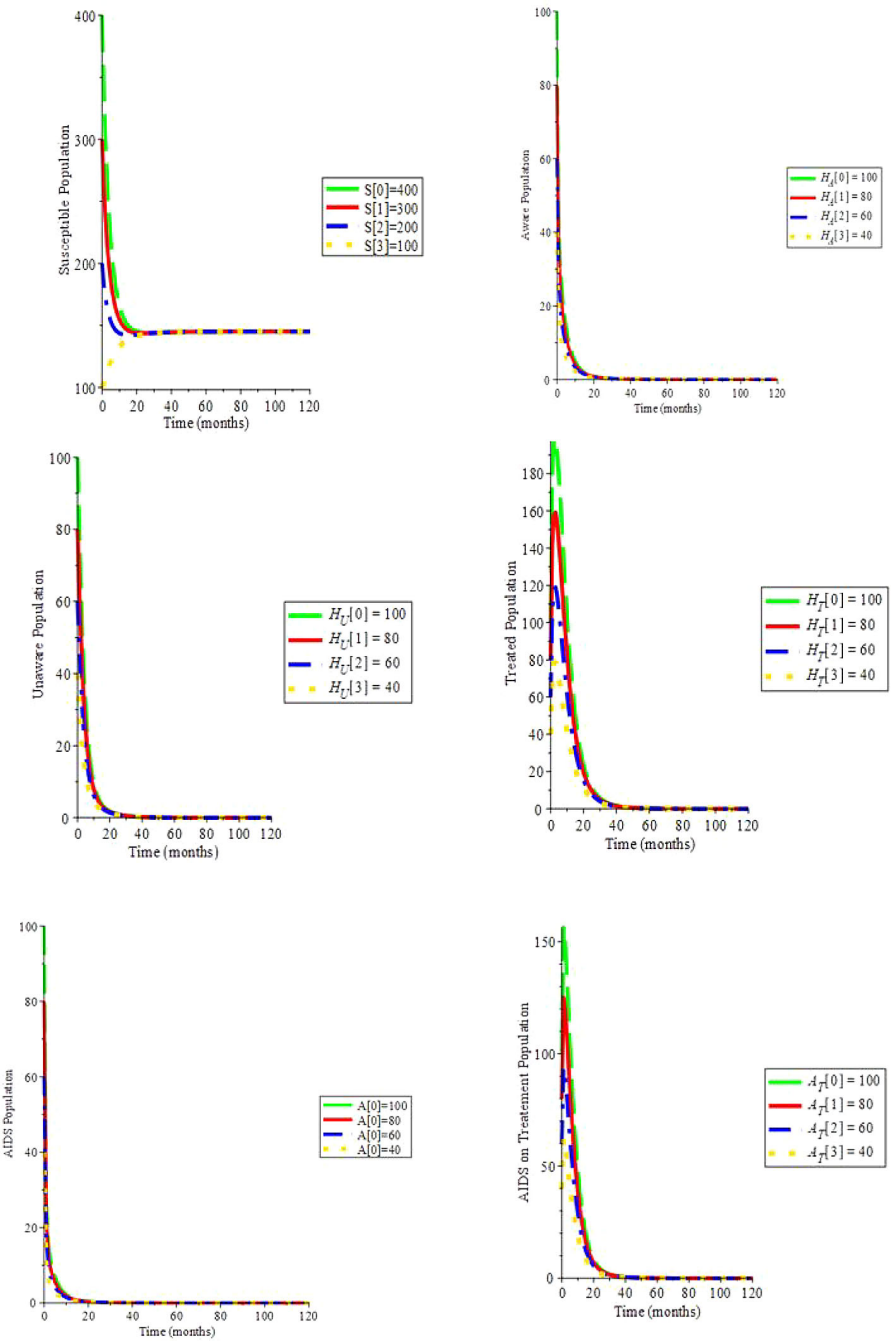
(Simulation 1) if *R*_0_ < 1, the infection-free equilibrium is asymptotically stable.

To affirm the model’s theoretical prognosis, simulation studies of the system (1) are run with the estimated parameter values listed below:

Simulation 1. Take into account the parametric data in Table 2 *c* = 3, *ψ* = 0, *ξ* = 0, *β*_1_ = 0.050, *β*_2_ = 0.055, *β*_3_ = 0.060, *μ* = 0.2, Λ = 29, *α* = 0.7, *ρ* = 0.322, *ζ* = 0.02, *υ*_1_ = 0.0169, *υ*_2_ = 0.0169, *θ*_1_ = 1.6949, *θ*_2_ = 1.6949, *d _a_* = 0.0333: Hence, *R*_0_ = 0.698 and the infection-free equilibrium is (145.000;0;0;0;0;0): We can see in Figure 2 that by changing the initial values, the solution trajectories intersect to (145.00;0;0;0;0;0): This confirms the fact that if *R*_0_ < 1, the virus-free equilibrium is globally asymptotically stable:

**Table 2.** Definition of Parameters values for the HIV model.

Parameters	Description	Parameters value	Source
Λ	Recruitment rate	29 *yr* ^−^1	^3^
*ζ*	Rate of newborns infected with HIV	0.02	[Assumed]
*c*	Contact rate	3 *patners*/ *yr*	^3^
*β _i_*, *i* = 1, 2, 3	Transmission rate for the infective HIV and AIDS	[0.050, 0.055, 0.060]	Assumed
*μ*	Natural mortality	0.2	[Assumed]
*α*	Testing rate	0.7	[Assumed]
*ρ*	Progression rate from Unaware HIV to AIDS	0.322	[Assumed]
*υ _i_*, *i* = 1, 2	HIV and AIDS defaulters from treatment	0.0169	^51^
*θ*_1_, *i* = 1, 2	HIV and AIDS treatment rate	1.6949	^27^
*d _a_*	Mortality due to AIDS	0.0333	[Assumed]
*ψ*	condom effectiveness	[0,1]	[Assumed]
*ξ*	condom usage	[0,1]	[Assumed]

**Figure 3.**
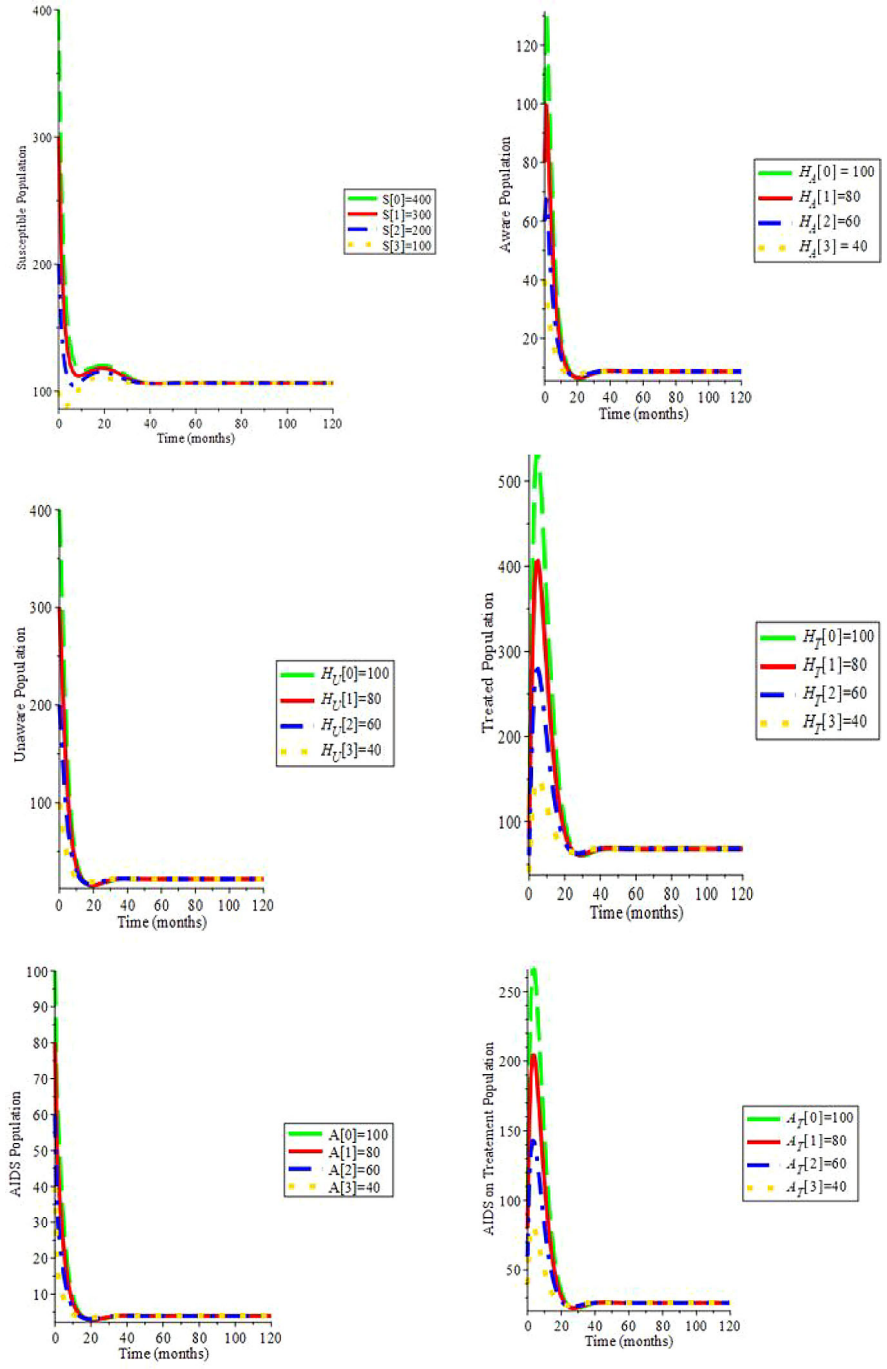
(Simulation 2) If *R*_0_ > 1, the endemic stability is asymptotically stable.

Simulation 2. Let *c* = 6, *ψ* = 0, *ξ* = 0, *β*_1_ = 0.080, *β*_2_ = 0.085, *β*_3_ = 0.090, *μ* = 0.2, Λ = 29, *α* = 0.7, *ρ* = 0.322, *ζ* = 0.02, *υ*_1_ = 0.0169, *υ*_2_ = 0.0169, *θ*_1_ = 1.6949, *θ*_2_ = 1.6949, *d _a_* = 0.0333: Hence, *R*_0_ = 2.197. Moreover, the endemic equilibrium is (64.197;13.225;5.251;41.035;2.348;15.905): We can see in Figure 3 that by changing the initial conditions, the solution trajectories intersect to (64.197;13.225;5.251;41.035; 2.348;15.905): This proves Theorem 5: if *R*_0_ > 1, the endemic stability is globally stable.

**Figure 4.**
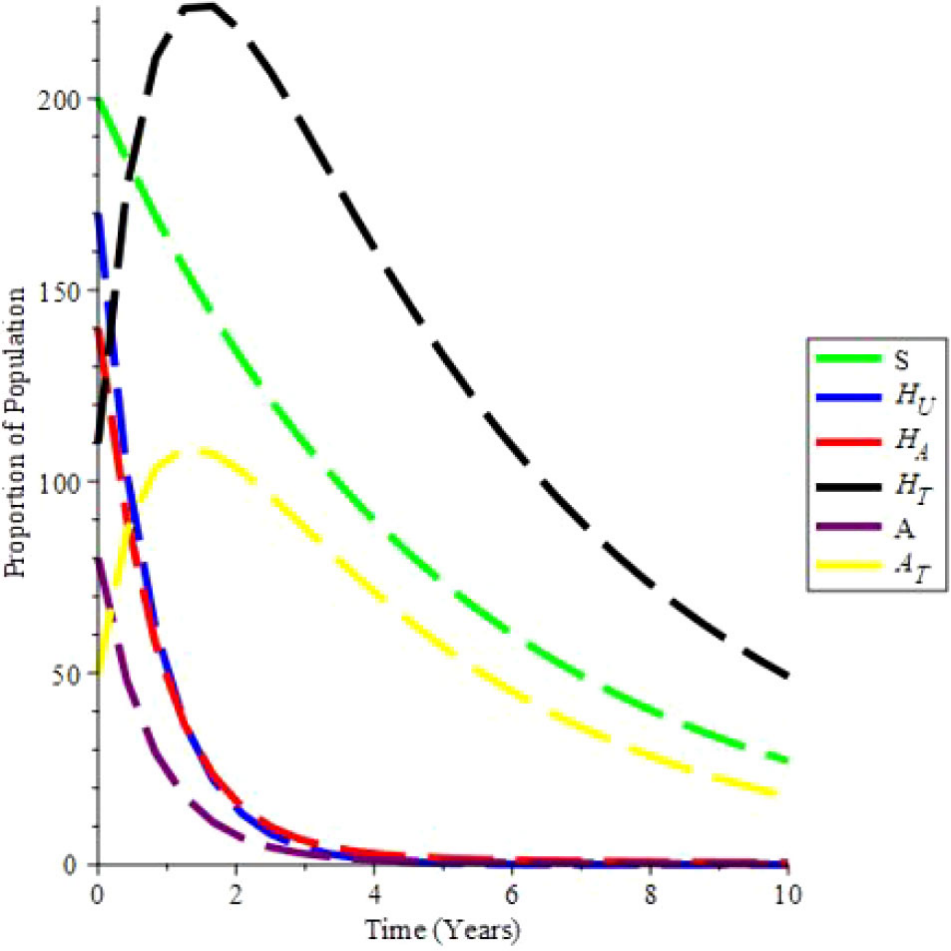
(Simulation 3) Take *ψ* = 1 and *ξ* = 1,to check the impact of condom use and effectiveness on the population when there’s no contact.

Simulation 3 depicts the distribution of individual proportions over time in various classes where there are no new infected children *ζ* or recruitment Λ, and contact *c* i.e. taking *c* = 0, *ζ* = 0, Λ = 0 when *ψ* = 1 and *ξ* = 1, (condom usage and effectiveness)i.e when there is full protection keeping every other values at endemic equilibrum constant, the value of *R*_0_ = 0.

The impact of perinatal transmission in the system, i.e. the incidence of new recruits of infected children directly into the infective group, is pointedly demonstrated in simulation 4.

**Figure 5.**
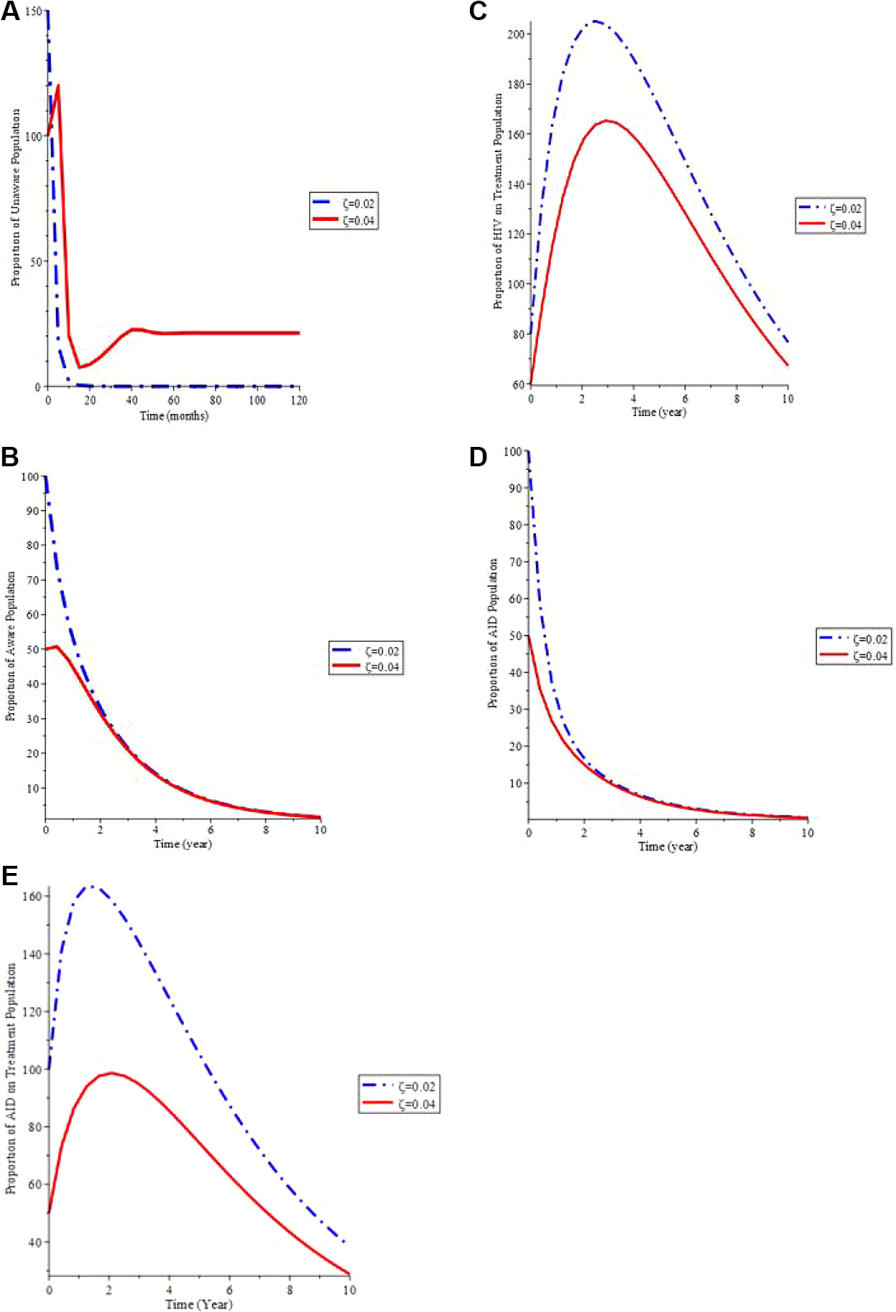
(Simulation 4) Variation in the infected individual for different *ζ* values. A. Variation of Unaware HIV population for different values of *ζ*. B. Variation of Aware HIV population for different values of *ζ*. C. Variation of HIV on Treatment population for different values of *ζ*. D. Variation of AIDS population for different values of *ζ*. E. Variation of AIDS on Treatment population for differnet values of *ζ*.

Figure 5(a) shows that as the proportion of infected newborns ( *ζ*) rises, so does the proportion of the general population who is unaware. Figure 5(b) shows that increasing the value of ( *ζ*) causes the proportion of the AIDS population to decrease over time, then raise until it reaches its stable state. As a result, if newborns infected with the virus are treated, the total infective group will be better controlled, minimizing the AIDS individuals. Figure 5(c) shows that as the number of infected children born rises, so does the treated populace.

The effect of defaulters on treatment lost to follow-up in the model is examined in simulation 5.

**Figure 6.**
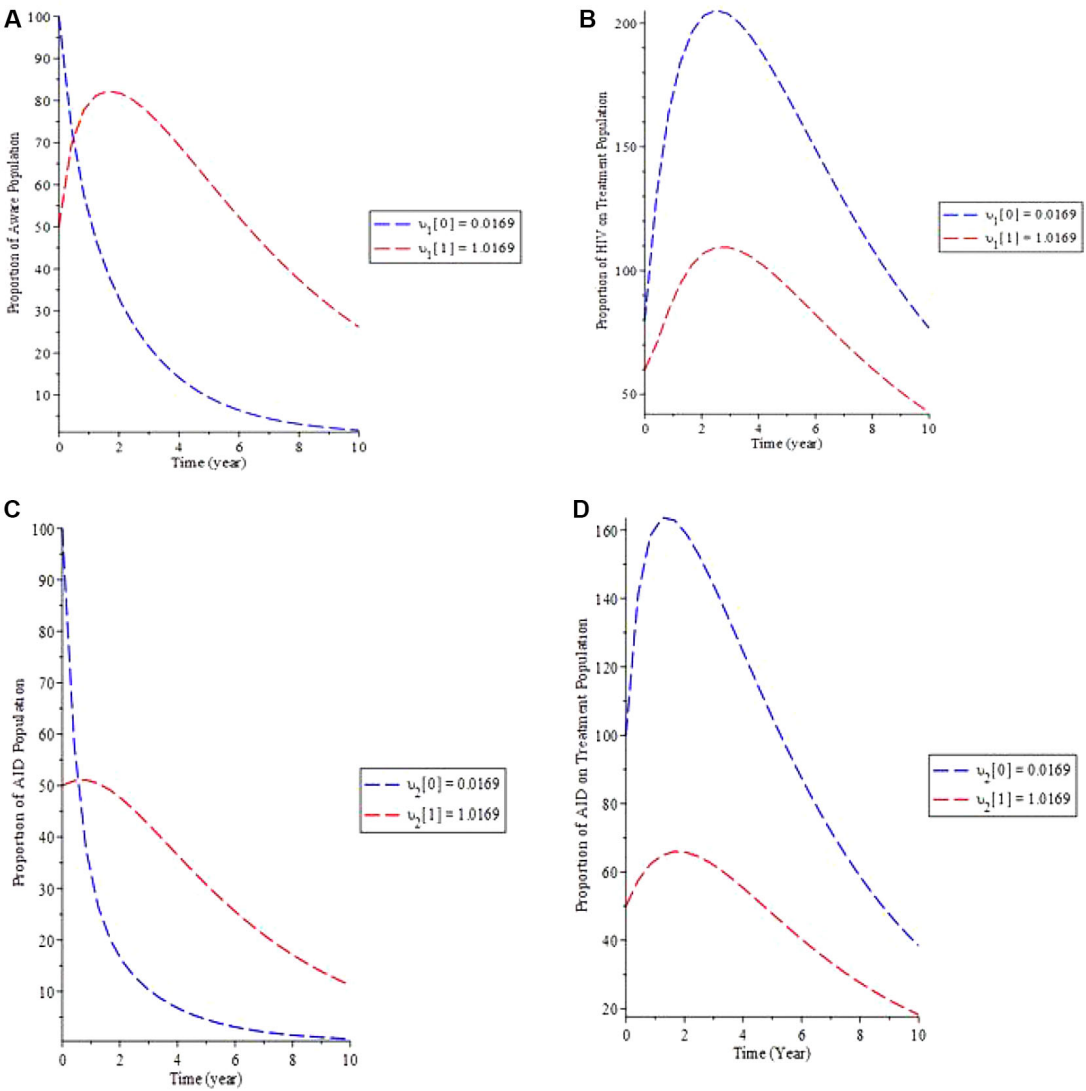
(Simulation 5) Variation of the infected individual for different fallout, *υ* values. A. Variation of HIV Aware population for different values of *υ*. B. Variation of HIV on Treatment population for different values of *υ*. C. Variation of AIDS population for different values of *υ*. D. Variation of AIDS on Treatment population for different values of *υ*.

Figure 6(a) shows that as the rate of defaulters ( *υ*) increases, so does the proportion of the population that is aware, whereas the proportion of HIV patients on treatment decreases (b). Figure 6(c) shows how increasing upsilon causes the proportion of the AIDS population to increase over time while decreasing the proportion of the AIDS population on treatment until equilibrium is reached. As a result, if the HIV-aware infected population follows adheres therapy, the infectious individual as a whole would then remain under control, lowering the HIV-aware and AIDS number of individuals.

The increasing effect of testing and treatment on the model is examined in simulation 6.

**Figure 7.**
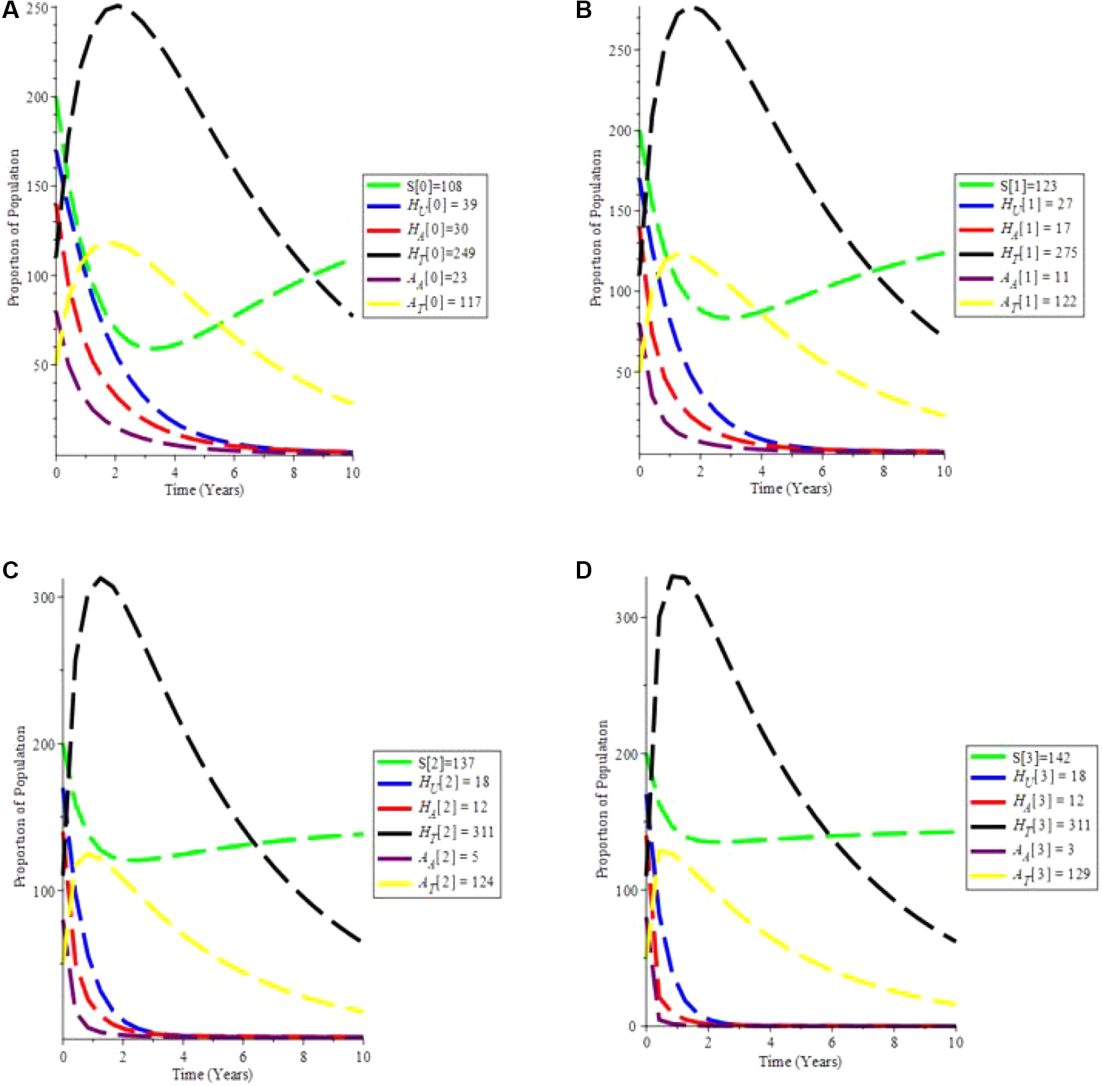
(Simulation 6) Proportion of different Population at the increased values of *α* and *θ*. A. Proporation of Population when *α* = 0.7 and *θ* = 1.6949. B. Proporation of Population when *α* = 0.9 and *θ* = 2.6949. C. Proporation of Population when *α* = 1.5 and *θ* = 4.6949. D. Proporation of Population when *α* = 1.9 and *θ* = 9.6949.

From Figure 7(a-d), it is observed that if testing rate and treatment rate is increase,the unaware HIV decrease,while aware HIV and AIDS individual decrease with time due to treatment. Furthermore, the susceptible individual increases, and as treatment increases, so does the population of HIV and AIDS patients on treatment. As a result, increasing HIV screening and treatment is the first procedure to UNAIDS’ 90-90-90 aspirations.

**Figure 8.**
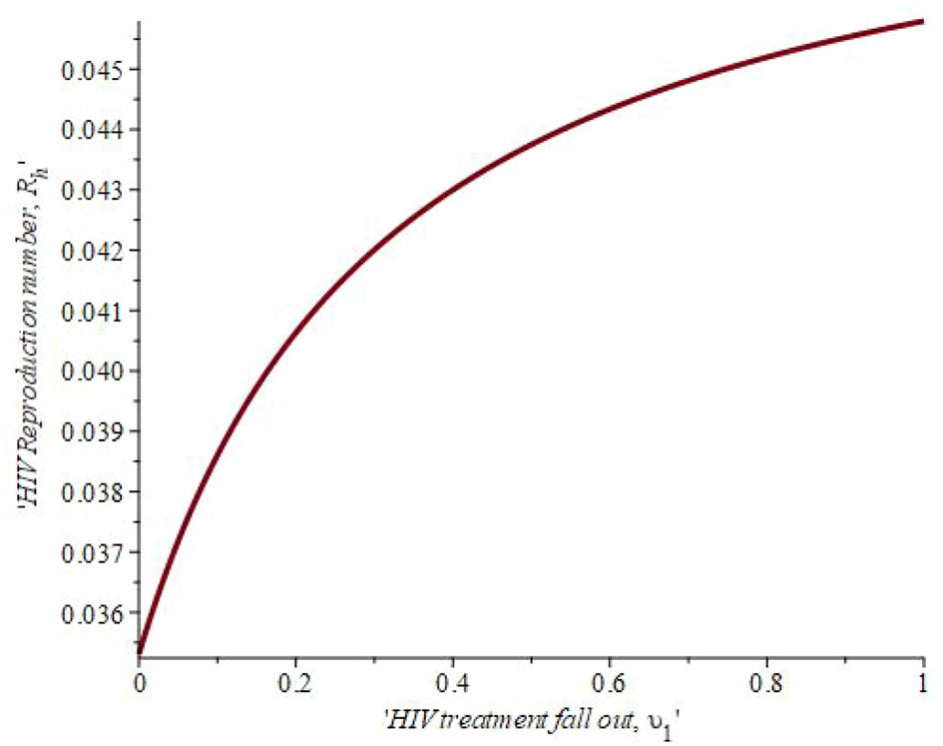
Impact of treatment fall out on HIV reproduction number.

Figure 8 shows the effect of treatment fall out on the reproduction number. When the number of infected individual on treatment that fallout is 19.8 percent then *R*_0_ = 0.041. The linear graphical representation also revealed that if 40.1 percent of the population drops out of treatment, the reproduction number rises to 0.043. This simply means that, as defaulters lost to follow-up increase, the reproduction number also increases. Hence, reducing high-risk habits, mainly through education, is the most effective way to reduce the overall number of HIV/AIDS patients.

## 6. Conclusions and recommendations

This study investigated the effect of testing and ART on the vertical and horizontal transmission dynamics of HIV/AIDS infection using an improved compartmental model and the dynamics theory of SI infectious diseases.

Reducing high-risk behaviours, primarily through education on the importance of HIV/AIDS status awareness and treatment adherence, is the best option for reducing the total number of HIV/AIDS patients.

Increased HIV testing is the first step toward UNAIDS’s 90-90-90 objectives, although many countries still face significant obstacles in attaining this goal. Early detection allows for prompt antiretroviral therapy, which lowers HIV viral load and hence slows the transmission of the virus. We believe that increasing HIV/AIDS diagnosis rates will increase the number of HIV/AIDS patients treated in the short term but decrease the number in the long term. WHO advises HIV self-testing as a complementary strategy, ^59^ which can improve the efficiency of HIV testing. ^60^

The current research showed that these intervention strategies are effective in combating the HIV/AIDS epidemic. This also emphasizes the need of behavioral and biologic therapies in preventing HIV transmission among pregnant women. This study has flaws, as well. First, statistics on drug resistance may be skewed because not all treated patients are tested early on, and secondly, homosexual transmission was not included in the model. Finally, certain characteristics were chosen on the basis of assumptions and may not really reflect reality.

In conclusion, the model implies that, in addition to HIV testing, behavioural and biologic strategies, effective condom use, and stringent adherence to ART are required for HIV prevention among individuals and pregnant women. Even in the face of medication resistance, ART and effective condom use can successfully limit the transmission of HIV. The 90-90-90 strategy may not be sufficient on its own to end the global HIV/AIDS outbreak.

## Data availability

The data in this article come from Mukandivire *et al*., 2010, Zu *et al*., 2016, Lu *et al*., 2020, and other assumed/estimated data.

## Software availability

Source code available from: https://github.com/OE-Abiodun/release/tag/v3.1.2

Archived source code at time of publication: https://doi.org/10.5281/zenodo.6894864. ^66^

License: GNU General Public License v3.0
