# Supplementary material for: Qualitative analysis of HIV and AIDS disease transmission: impact of awareness, testing and effective follow up
Source: F1000Res. 2023 Feb 28;11:1145. Originally published 2022 Oct 7. [Version 2] doi: 10.12688/f1000research.123693.2 (PMC9997043; doi:10.12688/f1000research.123693.2)
Supplement: Peer review history

# Peer review: Reviewer response for version 2

**DOI:** 10.5256/f1000research.143793.r164918

**Published:** 2023-03-13

**Authors:** Fatmawati Fatmawati

**Affiliations:** 1 Department of Mathematics, Faculty of Science and Technology, Universitas Airlangga, Surabaya, Indonesia

The authors have addressed most of the issues raised in my report, and improved the paper accordingly.

Is the work clearly and accurately presented and does it cite the current literature?

Partly

If applicable, is the statistical analysis and its interpretation appropriate?

Partly

Are all the source data underlying the results available to ensure full reproducibility?

No

Is the study design appropriate and is the work technically sound?

Partly

Are the conclusions drawn adequately supported by the results?

Partly

Are sufficient details of methods and analysis provided to allow replication by others?

Partly

Reviewer Expertise:

Mathematical modeling in life science, optimal control, fractional modelling.

I confirm that I have read this submission and believe that I have an appropriate level of expertise to confirm that it is of an acceptable scientific standard.

# Peer review: Reviewer response for version 2

**DOI:** 10.5256/f1000research.143793.r164917

**Published:** 2023-03-08

**Authors:** Adedapo Loyinmi

**Affiliations:** 1 Department of Mathematics, Tai Solarin University of Education, Ijebu ode, Nigeria

All observed lapses are corrected. The article is now fit for indexing.

Is the work clearly and accurately presented and does it cite the current literature?

Yes

If applicable, is the statistical analysis and its interpretation appropriate?

Yes

Are all the source data underlying the results available to ensure full reproducibility?

Yes

Is the study design appropriate and is the work technically sound?

Yes

Are the conclusions drawn adequately supported by the results?

Yes

Are sufficient details of methods and analysis provided to allow replication by others?

Yes

Reviewer Expertise:

Biological and computational Mathematics

I confirm that I have read this submission and believe that I have an appropriate level of expertise to confirm that it is of an acceptable scientific standard.

# Peer review: Reviewer response for version 1

**DOI:** 10.5256/f1000research.135825.r159237

**Published:** 2023-01-25

**Authors:** Ropo Ebenzer Ogunsakin

**Affiliations:** 1 Discipline of Public Health Medicine, School of Nursing & Public Health, College of Health Sciences, University of KwaZulu-Natal, Durban, South Africa

In this paper, the authors developed a new mathematical model for the transmission dynamics of HIV and AIDS and the model was rigorously analysed. The authors considered the impact of three major strategies (impact of awareness, testing and follow up) which are important in controlling the transmission dynamics of HIV/AIDS. The six- compartmental model of HIV/AID presented are: Susceptible, (S), representing people who prone to be infected with HIV in engage in risk habits/factors, those who are unaware of their HIV status (HU), those who become aware after testing (HA), those who are placed on treatment after becoming aware (HT); the AIDS individuals (AA) due to non-adherence to treatment and the AIDS on treatment population (AT). The author showed that the model is positive through a given region in their analyses and the reproduction number of the model was correctly worked out. The effects of the strategies were graphically represented in the numerical simulations. The title is peaks a volume to the context of the article.

 The work is well organized and approved for indexing. I have the following observation/comment on the paper:

1. The overall command of English looks good, however there are some grammatical errors in the paper. Also the authors should check for incomplete sentences in the paper.
2. Some representation can be made to reduce the size of the matrices as they are big or boxes should be adjusted.
3. It is necessary that the biological meaning of R0 be properly stated.
4. In the sensitivity section, the authors can as well placed the real values of the indices.
5. The conclusion looks good, the novelty of the work is discussed.

Is the work clearly and accurately presented and does it cite the current literature?

Yes

If applicable, is the statistical analysis and its interpretation appropriate?

Partly

Are all the source data underlying the results available to ensure full reproducibility?

Yes

Is the study design appropriate and is the work technically sound?

Yes

Are the conclusions drawn adequately supported by the results?

Yes

Are sufficient details of methods and analysis provided to allow replication by others?

Yes

Reviewer Expertise:

Statistician, Biological and Computational Mathematics

I confirm that I have read this submission and believe that I have an appropriate level of expertise to confirm that it is of an acceptable scientific standard.

# Peer review: Reviewer response for version 1

**DOI:** 10.5256/f1000research.135825.r158041

**Published:** 2023-01-05

**Authors:** Fatmawati Fatmawati

**Affiliations:** 1 Department of Mathematics, Faculty of Science and Technology, Universitas Airlangga, Surabaya, Indonesia

I have read this article. Some of my critical comments include:

1. Please explore the novelty of this article. What is the difference between this article and previous research that already exists.
2. I don't see the connection between the existence of the endemic equilibrium point and basic reproduction number (R0). Likewise to prove the stability of the disease-free equilibrium. The authors only mention that if R0 < 1 then the disease-free equilibrium is LAS, while depending on R0 cannot be proven exactly
3. Please also check for proof of global stability of the disease-free equilibrium.
4. The authors must proofread very carefully the language of the manuscript.
5. Carefully check in whole manuscript, dot, and comma after each equation.
6. Then numerical results need to be discussed in more details.
7. The conclusion sections should highlight the main findings of this study.
8. References should be up to date and must follow the style correctly.

Is the work clearly and accurately presented and does it cite the current literature?

Partly

If applicable, is the statistical analysis and its interpretation appropriate?

Partly

Are all the source data underlying the results available to ensure full reproducibility?

No

Is the study design appropriate and is the work technically sound?

Partly

Are the conclusions drawn adequately supported by the results?

Partly

Are sufficient details of methods and analysis provided to allow replication by others?

Partly

Reviewer Expertise:

Mathematical modeling in life science, optimal control, fractional modelling.

I confirm that I have read this submission and believe that I have an appropriate level of expertise to state that I do not consider it to be of an acceptable scientific standard, for reasons outlined above.

# Peer review: Reviewer response for version 1

**DOI:** 10.5256/f1000research.135825.r152688

**Published:** 2022-11-18

**Authors:** Adedapo Loyinmi

**Affiliations:** 1 Department of Mathematics, Tai Solarin University of Education, Ijebu ode, Nigeria

The article proposed a mathematical model for the transmission dynamics of HIV and AIDS considering three control/preventive strategies: the impact of awareness, testing and follow up. The work presented a six- compartmental model of HIV/AID which are Susceptible, ( *S*), representing people who are likely to become infected with HIV; Unaware HIV infective, ( *HU*), Aware HIV infective ( *HA*), Treated HIV infective, ( *HT*); AIDS individuals ( *AA*) and AIDS on treatment individuals ( *AT*). Analysis of the model showed that the model is positively invariant. Also, the reproduction number of the model was accurately worked out. The stability of the model showed that the disease free and endemic equilibrium is stable if necessary conditions are satisfied. The numerical simulations graphically showed the effect of the control strategies. **The work is well organized and suitable for indexing**.

 However, a few issues should be addressed:

1. The manuscript needs to be properly arranged as some of the matrices are too big. The boxes should be adjusted.
2. Under stability analysis of the DFE, the R0 is not clearly shown. Since conclusion depends on the value of R0, it is necessary that R0 is properly substituted in the equation.
3. In the sensitivity section, the sensitivity value need to be presented in order to know how sensitive each parameters are. The + and - signs are not enough
4. Graphs are not bold enough.
5. Revisit the conclusion under stability of DFE to drive home your findings
6. In figure 5, I suggest that more than two values of ***ζ*** ****be used **.**
7. In the conclusion, the novelty of the work is not elaborately discussed. Please rewrite this part to show what the contribution to knowledge is.
8. There should be a paragraph discussing earlier work on diseases such as Ebola, covid-19 and the likes threatening global health. Such articles like, but not limited to 'qualitative analysis and dynamical behaviour of a Lassa haemorrhagic fever model with exposed rodents and saturated incidence rate' would help.

Is the work clearly and accurately presented and does it cite the current literature?

Yes

If applicable, is the statistical analysis and its interpretation appropriate?

Yes

Are all the source data underlying the results available to ensure full reproducibility?

Yes

Is the study design appropriate and is the work technically sound?

Yes

Are the conclusions drawn adequately supported by the results?

Yes

Are sufficient details of methods and analysis provided to allow replication by others?

Yes

Reviewer Expertise:

Biological and computational Mathematics

I confirm that I have read this submission and believe that I have an appropriate level of expertise to confirm that it is of an acceptable scientific standard, however I have significant reservations, as outlined above.
